# Pharmacologic therapies of ARDS: From natural herb to nanomedicine

**DOI:** 10.3389/fphar.2022.930593

**Published:** 2022-10-28

**Authors:** Linlin Meng, Ximing Liao, Yuanyuan Wang, Liangzhi Chen, Wei Gao, Muyun Wang, Huiling Dai, Na Yan, Yixuan Gao, Xu Wu, Kun Wang, Qinghua Liu

**Affiliations:** ^1^ Shandong University of Traditional Chinese Medicine, Jinan, Shandong, China; ^2^ Department of Critical Care Medicine, Shanghai East Hospital, School of medicine, Tongji University, China; ^3^ Department of Gynecology, Shandong Provincial Hospital Affiliated to Shandong First Medical University, Jinan, Shandong, China; ^4^ Department of Respiratory and Critical Care Medicine, The First Affiliated Hospital of Anhui Medical University, Hefei, Anhui, China

**Keywords:** acute lung injury, acute respiratory distress syndrome, pharmacology, nanomedicine, drug delivery, natural herb

## Abstract

Acute respiratory distress syndrome (ARDS) is a common critical illness in respiratory care units with a huge public health burden. Despite tremendous advances in the prevention and treatment of ARDS, it remains the main cause of intensive care unit (ICU) management, and the mortality rate of ARDS remains unacceptably high. The poor performance of ARDS is closely related to its heterogeneous clinical syndrome caused by complicated pathophysiology. Based on the different pathophysiology phases, drugs, protective mechanical ventilation, conservative fluid therapy, and other treatment have been developed to serve as the ARDS therapeutic methods. In recent years, there has been a rapid development in nanomedicine, in which nanoparticles as drug delivery vehicles have been extensively studied in the treatment of ARDS. This study provides an overview of pharmacologic therapies for ARDS, including conventional drugs, natural medicine therapy, and nanomedicine. Particularly, we discuss the unique mechanism and strength of nanomedicine which may provide great promises in treating ARDS in the future.

## Introduction

The term “acute respiratory distress syndrome” (ARDS) was first proposed by Ashbaugh et al. (1967), who noticed that a class of patients appeared with similar clinical manifestations (e.g., tachypnea, hypoxemia, and loss of compliance) after sepsis, trauma, and other clinical disorders. Since then, the mask of this life-threatening condition has gradually been revealed by generations of investigators and reached a consensus in 2012 Berlin. According to the Berlin definition proposed in 2012, ARDS is clinically recognized by acute onset of hypoxemia (partial pressure of oxygen to fraction of inspiration oxygen, PaO2/FiO2 ≦ 300 mmHg) with bilateral opacities on chest radiography or computer tomography (CT) within 7 days after known pulmonary (pneumonia, aspiration) or non-pulmonary (sepsis and trauma) insults (Ferguson et al., 2012). According to statistics, the global incidence of ARDS is about 7–34 cases per 100,000 people per year (Luhr et al., 1999; Bersten et al., 2002; Kaku et al., 2019). With the improvement of respiratory critical care management, especially the standardized use of mechanical ventilation, the mortality of ARDS has dropped to 26% and 35%, far lower than the originally reported 60% (Stapleton et al., 2005; Mutlu et al., 2006; Dellinger, 2010). Unfortunately, ARDS is still a major reason for intensive care unit (ICU) admission and mechanical ventilation, a huge public health burden globally (Mutlu et al., 2006; Bellani et al., 2016). Meanwhile, since the current pandemic of severe acute respiratory syndrome coronavirus (SARS-COV2) in 2019, the 2019 Coronavirus Disease (COVID-19) has already become a leading cause of acute lung injury (ALI) and ARDS. Data in 2020 show that 42% of COVID-19 pneumonia patients will progress to ARDS, of which 61%–81% require intensive care (Wu et al., 2020). Though this rate decreases due to widespread vaccination, the in-depth study of virus features, and the recent mutation of the SARS-2 strain, it remains a cause for caution during the pandemic. Therefore, there is an urgent need to improve ARDS management and find effective treatment approaches.

The poor performance of ARDS is closely related to its heterogeneous clinical syndrome caused by complicated pathophysiology, which mainly includes alveolar endothelial and epithelial injury, alveolar permeability dysfunction, and lung inflammation dysregulation, thus leading to decreased lung volume and compliance and an imbalance of ventilation/blood flow (Meyer et al., 2021). Based on the different pathophysiology phases, drugs, protective mechanical ventilation, conservative fluid therapy, and other treatment have been developed to serve as the ARDS therapeutic methods. Numerous pharmaceutical industries and research teams have garnered significant attention for drug treatment of ARDS to reduce its mortality and improve prognosis.

In this review, we first briefly discuss the pathophysiology of ARDS and demonstrate its underlying treatment target. Then, we summarize present advances in pharmacologic therapies in ARDS, including conventional drugs, natural medicine therapy, and nanomedicine. Particularly, we discuss the unique mechanism of nanodevices which may provide great promises in treating ARDS in the future.

## Pathophysiology of ARDS

**FIGURE 1:**
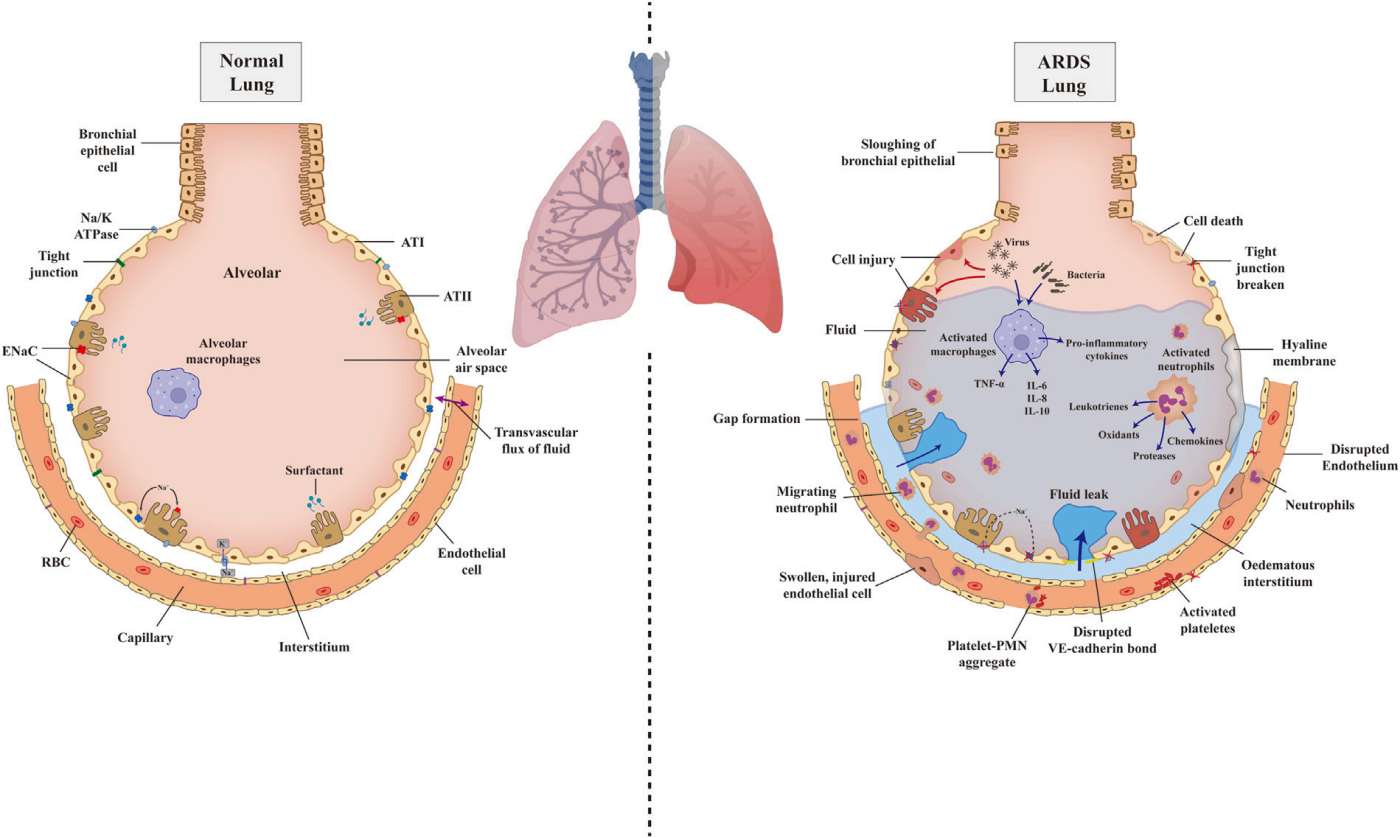
The pathophysiology of normal lung and ARDS injured lung. Schematic illustration depicting pathophysiology of the normal lung (left) progress to ARDS lung (right) due to the attack of pathogens and illustrating the details of related mechanisms. AT, alveolar type cell; ENaC, epithelium sodium channel; RBC, red blood cell; TNF-α, tumor necrosis factor-α; IL, interlukin; PMN, polymorphonuclear cells; VE-cadherin, vascular endothelial cadherin.

In normal lung, endothelial cells and epithelial cells, such as alveolar type cells (ATⅠ and ATⅡ), combine with plasma membrane structures to compose a single-layer barrier through tight junctions and adherence. This intact barrier can selectively allow the passage of gas, solutes, and a certain amount of fluids (Bhattacharya and Matthay, 2013). Sodium channel (ENaC) and Na/K ATPase express on ATⅠ and ATⅡ transport fluid from the alveolar into the lung interstitium pass through the intact alveolar epithelial layer; fluid is then drained by lymphatics or reabsorbed into the vasculature, named alveolar fluid clearance (AFC) (Matthay and Wiener-Kronish, 1990; Ware and Matthay, 2001; Johnson et al., 2002) (Figure 1).

However, in ARDS or ALI, bacteria or other cell-injury associated danger-associated molecular patterns (DAMPs) bind to Toll-like receptors (TLR), which are expressed on alveolar macrophages and epithelial cells and will induce the innate immune system activation (Opitz et al., 2010). However, a virus, such as SARS-CoV-2, combines with angiotensin-converting enzyme 2 (ACE2), subsequently binds to Toll-like receptors, and shows the same effect (Li et al., 2003). Excessive and dysregulated inflammation directly attack the alveolar, leading to endothelial and epithelial damage. Macrophage and other sources of cytokines, tumor necrosis factor-α (TNF-α), and leukocyte concentrations in the lungs destabilize the vascular endothelial cadherin (VE-cadherin) bonds, causing increased endothelial permeability and inducing the accumulation of alveolar fluid (Corada et al., 1999; Schulte et al., 2011). Furthermore, increased alveolar-capillary permeability induces the recruitment of platelets, proteins, polymorphonuclear cells (PMN), red blood cells, and effector T cells, aggravating the epithelial injury (Fe In et al., 1979; Bachofen and Weibel, 1982). The inter-cellular endothelial cadherin (E-cadherin) junctions of epithelial are disrupted by migration neutrophils, leading to denudation and apoptosis, ultimately resulting in epithelial breakage and increasing permeability (Ginzberg et al., 2004). On the one hand, after alveolar injury, the capacity of AFC is reduced due to epithelial ENaC and the Na/K-ATPase less efficiently, as well as osmotic gradient abnormality. On the other hand, the damaged epithelial cell loses the function of surfactant production, which brings impeded alveolar patency and alveolar collapse (Mac Sweeney and McAuley, 2016). Once pulmonary edema fluid accumulates, in combination with the development of hyaline membranes, pulmonary compliance decreases and gas exchange is impaired, causing hypoxemia, hypercapnia, ventilation/perfusion (V/P) mismatch, and ultimately acute respiratory failure (Matthay et al., 2012) (Figure 1).

According to the above pathophysiological mechanisms, different drugs have been developed to treat diverse targets in the pathogenesis process. Therefore, we will introduce the mechanism of these drugs and discuss their current therapeutic application progress in the following sections (Table 1).

**TABLE 1:** Traditional medicines for the treatment of ARDS.

Drugs	Targets	Status	Model	Dose	References
Class of biochemical agents					
Losartan	LOX-1	Preclinical	Rat model	8 mg/kg	Deng et al. (2015)
	JAK2/STATs	Preclinical	Rat model	5, 15, 30 mg/kg	Li N et al. (2017)
Sivelestat	NE	Clinical	Patients	0.2 mg/kg/h	Tagami et al. (2014)
					Miyoshi et al. (2013)
Palifermin	KGF receptor	Clinical	Patients	60 μg/kg	Shyamsundar et al. (2014)
Palifermin	SP-D	Preclinical	Human *in vivo* short-term model	60 μg/kg	Mcauley et al. (2017)
Acetylsalicylic acid	TX-A2	Preclinical	Murine model	1 mg/g	Singbartl and Ley (2007)
15-Epi-LXA4	β2-Integrin	Preclinical	Murine model, human neutrophils	0.03–2 mM in cells, 200 mg/kg in mice	Kebir et al. (2009)
	Mac-1				
	AT-RvD1	Preclinical	Murine model	0.5–0.05 μg/kg	Cox et al. (2015)
	TX-B2	Preclinical	EVLP model	1200 mg/day, 75 mg/day	Hamid et al. (2017)
	—	Clinical	Patients	325 mg loading dose followed by 81 mg/d	Kor et al. (2016)
Simvastatin	—	Preclinical	Murine model	5 or 20 mg/kg	Jacobson et al. (2005)
	—	Preclinical	Human MDMs, human neutrophils	40 or 80 mg	Shyamsundar et al. (2009)
	—	Clinical	Patients	80 mg	Mcauley et al. (2014b)
Rosuvastatin	—	Clinical	Patients	40 mg loading dose followed by 20 mg/d	Truwit et al. (2014)
Atorvastatin	—	Clinical	Patients	20 mg	Investigators (2022)
Cisatracurium	nAChRα1	Clinical	Patients	Bolus of 50 mg, followed by 5 μg/kg/min	Gainnier et al. (2004)
	nAChRα1	Clinical	Patients	Bolus of 15 mg, followed by 37 0.5 mg/h	Papazian et al. (2010)
Class of cytokine-based therapy					
Interferon-β-1a (FP-1201)	CD73	Clinical	Patients	1000 IU/ml	Geoff et al. (2014)
Interferon-β-1a (SNG001)	—	Clinical	Patients	6 million international units	Monk et al. (2021)
TNFα-MAb	TNF-α	Preclinical	Pig model	5 mg/kg	Mullen et al. (1993)
Etanercept	TNFRII	Preclinical	Rat model, rat primary alveolar type II cells	1 mg/kg in rats, 1 μg/ml in cells	Guthmann et al. (2009)
GSK1995057	TNFRI	Preclinical	HMVEC-L, monkey model	10 nM in cells, 0.043, 0.45, 4.7 mg in monkeys	Proudfoot et al. (2018)
Tocilizumab	Anti-IL-6	Clinical	Patients	400–800 mg (depending on weight)	Lewis (2021)
	Anti-IL-6	Clinical	Patients	8 mg per kg of body weight	Rosas et al. (2021)
Canakinumab	anti-IL-1β	Clinical	Patients	Two doses of 150 mg (or 2 mg/kg for participants weighing ≤40 kg)	Mastroianni et al. (2021)
IL-8 antibody	anti-IL-8	Preclinical	Rabbits	2.5 mg/rabbit	Bao et al. (2010)
Class of inhalation agents					
Salbutamol	β-2R	Clinical	Patients	15 μg/kg/h	Perkins et al. (2006)
					Gao et al. (2012)
	β-2R	Clinical	Patients	100 mg twice daily	Perkins et al. (2014)
Unfractionated heparin	NF-κB	Preclinical	Rat model	100, 300 U/kg	Li et al. (2013)
Heparin sodium	—	Clinical	Patients	5, 10, 20, 40 million international units	Dixon et al. (2008)
	—	Clinical	Patients	2.5 million international units	Dixon et al. (2010)
	—	Clinical	Patients	5 million international units	Dixon et al. (2016)
Enoxaparin	—	Clinical	Patients	30, 40 mg	Rentsch et al. (2021)
Calfactant	—	Clinical	Patients	80 ml/m^2^	Willson et al. (1999)
					Willson et al. (2005a)
	—	Clinical	Patients	30 mg/cm	Willson et al. (2013)
					Willson et al. (2005b)
Nitric oxide	cGMP	Clinical	Patients	5–20 parts per million	Rossaint et al. (1993)
					Michael et al. (1998)
	cGMP	Clinical	Patients	5 parts per million	Taylor et al. (2004)
Class of corticosteroid					
Methylprednisolone	—	Clinical	Patients	30 mg/kg	Bernard et al. (1987)
					Luce et al. (1988)
	—	Clinical	Patients	1 mg/kg	Meduri et al. (2007)
Dexamethasone	—	Clinical	Patients	6 mg	Group (2021)
Class of cell-based therapy					
Human mesenchymal stem	—	Preclinical	Rat model, U937 monocytic/macrophage cell line	2, 5, 10, 20 million/kg in rats and cells	Devaney et al. (2015)
	AKT	Preclinical	*Ex vivo* perfused human lung model, human blood monocytes	100 μl in cells	Lee et al. (2013)
	—	Preclinical	Human alveolar epithelial cells, murine model	5 × 10^5^ cells in 100 μl in mice and cells	Yan et al. (2016)
Mice mesenchymal stem	—	Preclinical	Murine model	1 × 10^5^ cells	Chan et al. (2016)
Human mesenchymal stem	—	Clinical	Patients	1, 10 million cells/kg PBW	Wilson et al. (2015)
Class of natural herb therapy					
Honokiol	—	Preclinical	Murine model	2.5, 5 mg/kg	Weng et al. (2011)
	Sirt3/AMPK	Preclinical	Murine model, HPMECs	2.5, 5 mg/kg in mice, 1.25, 5 μM in cells	Lan et al. (2018)
	Nrf2	Preclinical	Rat model, BEAS-2B cell line	1.25, 2.5, 5 mg/kg in rats, 12.5, 25, 50 μM in cells	Liu S et al. (2021)
Isoforskolin	—	Preclinical	Rat model, human mononuclear leukocyte cell line	5, 10, 20 mg/kg in rats, 100, 200 μM in cells	Yang et al. (2011)
Caffeic acid phenethyl ester	—	Preclinical	Rat model	10 μmol/kg	Koksel et al. (2006)
	—	Preclinical	Rat model	50 μmol/kg	Fidan et al. (2007)
	MD2	Preclinical	Murine model, RAW264.7 and 293T cell lines	1% in mice, 1, 5, 10 μM in cells	So et al. (2013)
	MD2	Preclinical	Murine model, mice peritoneal macrophage	15 mg/kg in mice, 10 μM in cells	Chen et al. (2018)
*Callicarpa japonica* Thunb.	—	Preclinical	Murine model, RAW264.7 cell line	30 mg/kg in mice, 5, 10, 20, 40 μg/ml in cells	Shin et al. (2015)
Acteoside	NF-κB	Preclinical	Murine model, A549 cell line	30, 60 mg/kg, 1, 10, 100 μM in cells	Jing et al. (2015)
Forsythoside B	—	Preclinical	Rat model, RAW264.7 cell line	40 mg/kg in rats, 3 and 9 μmol in cells	Jiang et al. (2012)
Salidroside	—	Preclinical	Murine model	120 mg/kg	Guan et al. (2012)
	Caveolin-1	Preclinical	Rat model, A549 cell line	20, 40 mg/kg in rats, 10, 20, 40, 80, and 160 μM in cells	Jingyan et al. (2017)
	SIRT1	Preclinical	Murine model, MLVECs	50 mg/kg in mice, 50 μM in cells	Wang et al. (2017)
	SIRT1	Preclinical	Murine model, RAW264.7 cell line	20, 40 mg/kg in mice, 30, 60, and 120 μM in cells	Lan et al. (2017)
Hydroxysafflor yellow A	TLR4	Preclinical	Murine model	40, 80 and 120 mg/kg	Liu M et al. (2014)
Xuebijing	—	Preclinical	Rabbit model	10 ml/kg	Ji et al. (2016)
Eriodictyol	Nrf2	Preclinical	Murine model	30 mg/kg	Zhu et al. (2015)
	—	Preclinical	Murine model	20, 40, 80 mg/kg	Wang X et al. (2020)
Asperuloside	NF-κB/AMPK	Preclinical	Murine model, RAW264.7 cell line	20, 40, 80 mg/kg in mice, 0–80 mg/L in cells	Qiu Y et al. (2016)
	NF-κB/AMPK	Preclinical	RAW264.7 cell line	0, 50, 100, 200 μg/ml	He et al. (2018)
Paeoniflorin	—	Preclinical	Murine model	5, 10, 20 mg/kg	Cao et al. (2011)
	—	Preclinical	Murine model	50, 100 mg/kg	Zhou et al. (2011)
	αvβ3/TGF-β1	Preclinical	Murine model	50, 100 mg/kg	Yu et al. (2021)
Curcumin	SIRT1	Preclinical	Murine model, RAW264.7 cell line	100, 200 mg/kg, 20, 40, 80 μM	Wang et al. (2021)
	—	Preclinical	A549 cell line	10, 20, 50, 100 μM	Hu et al. (2020)
	—	Preclinical	Murine model	150 mg/kg	Bansal and Chhibber (2010)
	—	Preclinical	Murine model, mice primary BMDMs	50 mg/kg in mice, 20 μM in cells	Xu et al. (2015)
	—	Preclinical	Murine model	5 mg/kg	Tyagi et al. (2014)
	—	Preclinical	Murine model	10 mg/kg	Kumari et al. (2015)
	AMPK	Preclinical	Murine model, RAW264.7 cell line	200 mg/kg in mice, 0–30 µM in cells	Kim et al. (2016)
	—	Preclinical	Murine model, mice CD4 naive T cells	20 mg/ml in mice, 100 ng/ml in cells	Chai et al. (2020)

LOX-1, low-density lipoprotein receptor 1; NF-κB, nuclear factor kappa light chain enhancer of activated B cells; JAK2, Janus kinase 2; STAT, signal transducer and activator of transcription proteins; NE, neutrophil elastase; TX-A2, thromboxane-A2; 15-epi-LXA4, human 15-epi-oxolipin-A4; AT-RvD1, aspirin-triggered resolvin D1; nAChRα1, nicotinic acetylcholine receptor α1; TNFR, tumor necrosis factor receptor; β-2R, β-2 receptor; SIRT, sirtuins; AMPK, amp activated protein kinase; Nrf2, nuclear factor erythroid 2-related factor 2; cGMP, cyclic guanosine monophosphate; MD-2, myeloid differentiation protein 2; TLR4, toll-like receptor 4; TGF-β1, transforming growth factor-β1; αvβ3, integrin alpha V + beta 3; KGF, keratinocyte growth factor; SP, surfactant protein; AKT, protein kinase B; EVLP, *ex vivo* lung perfusion repair system; HMVEC-L, human pulmonary microvascular endothelial cells; PBW, predicted bodyweight; HPMECs, human peritoneal microvascular endothelial cells; MLVECs, mouse lung vascular endothelial cells; BMDM, bone marrow-derived macrophage; BEAS-2B, human normal lung epithelial cell; ALI, acute lung injury; ARDS, acute respiratory distress syndrome.

## Conventional drugs for the treatment of ARDS

### Biochemical agents

#### Targeting renin-angiotensin II-aldosterone system strategies

Renin-angiotensin-II-aldosterone system (RAAS) has long been recognized as a major regulatory mechanism of blood pressure and fluid/electrolyte balance, and angiotensin-converting enzyme (ACE), as a potent vasoconstrictor of RAAS, has been widely used as a target of antihypertensive drugs (Boehm and Nabel, 2002). Protein-rich fluid accumulation is often seen within the alveoli and interstitium during the exudative phase of ARDS. ACE and angiotensin II (ANG II) can affect the secretion of aldosterone to promote renal tubular reabsorption of fluid and sodium, thus moderating ARDS pathophysiology (Thomas et al., 2008).

Recent studies have shown that Ang II may increase microvascular permeability in the basal state (Victorino et al., 2002) and that invasively administered Ang II promotes the formation of pulmonary edema. However, applying appropriate doses of losartan, a common angiotensin-converting enzyme inhibitor (ACEI), prevents this form of pulmonary edema (Yamamoto et al., 1997). Losartan pretreatment also attenuated the lung injury caused by the high-expression AT1 receptor in the ventilator-induced ALI model (Yao et al., 2008). As for its underlying mechanisms, Deng et al. found that co-administration of losartan and lipopolysaccharide (LPS) could alleviate inflammation and cell apoptosis by suppressing the expression of low-density lipoprotein receptor 1 (LOX-1) (Deng et al., 2015). In contrast, Li et al. discovered that pretreatment of losartan decreased the expression of nuclear factor kappa B (NF-κB) and the activation of the Janus kinase 2 (JAK2)/signal transducer and activator of transcription proteins (STATs) pathway, reducing the apoptotic ratio of cells through modulating phosphorylation and the leak of cytochrome C to the cytosol (Li C et al., 2017).

Imai et al. found that ACE2, a homolog of ACE with a negative effect on ANGII formation, along with angiotensin II- (AT2-) receptor protect against lung injury by aspiration or sepsis, whereas the ACE2 and AT1 receptor may promote the disease pathogenesis (Imai et al., 2010). In the influenza H5N1 virus-induced ARDS model, miR-200c-3p, one small non-coding RNA directly targets the 3′-UTR of ACE2, downregulating the ACE2 protein expression through an NF-κB-dependent manner. The inhibition of miR-200c-3p shows protective effects (Qiang et al., 2017). In a phase-II multicenter study completed in 2014, the infusion of GSK2586881, a recombinant version of the catalytic ectodomain of human ACE2 (rhACE2), was well tolerated among ARDS patients and able to decrease the level of Ang II while upregulating angiotensin (1-7), angiotensin (1–5), and surfactant protein D levels (Khan et al., 2017).

In addition, ACE2 was the main receptor for viral entry into host cells in a related study on COVID-19 (Hoffmann et al., 2020), and the quantitative level of ANG Ⅱ was positively correlated with viral titer (Liu et al., 2020). However, due to the lack of large preclinical studies, the specific mechanism is still unclear.

#### Neutrophil elastase inhibitor

Neutrophil elastase (NE) is one of the physiological proteolytic enzymes that attack the extracellular matrix, modulating inflammation and tissue remodeling. It could directly damage or exert pro-inflammatory and pro-apoptosis effects on the local tissue (Pham, 2006). During some intense inflammatory conditions such as ARDS, the balance between NE and its endogenous inhibitor has been disrupted (Polverino et al., 2017). Therefore, an exogenous NE inhibitor could be a solution for the epithelial and endothelial cell damage during ARDS.

Sivelestat, a specific NE inhibitor, has effectively attenuated lung damage by inhibiting inflammatory signaling pathways and affecting NE activity in the LPS-induced ALI model (Iba et al., 2006; Yuan et al., 2014). In mechanical ventilation (MV) associated with lung injury, sivelestat moderated the histopathological degree of lung damage and suppressed the expression of the inflammatory factors (Sakashita et al., 2007). According to several retrospectives or open-label studies, sivelestat improves ventilator-free days and 180-day survival in ARDS patients (Naoki et al., 2011; Miyoshi et al., 2013; Tagami et al., 2014). Early administration of sivelestat after esophagectomy surgery also shows the effect of ARDS prevention (Wang J et al., 2015). However, one large multicenter, double-blinded, randomized clinical trial of sivelestat was stopped due to the negative trend in long-term mortality rate, in which follow-up data showed neither effects on primary endpoints of ventilator-free days and 28-day all-cause mortality nor physiology and clinical outcomes (Zeiher et al., 2004).

In the subsequent meta-analysis enrolling eight clinical trials, sivelestat showed no difference in mortality within 28–30 days or MV days and was associated with a worse outcome for 180 days of mortality. However, a meta-analysis to evaluate the effect of sivelestat on the postoperative clinical course with esophagectomy cancer patients showed that sivelestat decreases the incidence of ALI/ARDS and MV duration. The incidence of pneumonia and the length of ICU and hospital stay were also decreased in the sivelestat group (Wang Z et al., 2015).

#### Keratinocyte growth factor

The alveolar epithelial cell injury is one of the initial steps of ARDS development (Thompson et al., 2017), leading to decreased AFC and alveolar edema. The restoration of alveolar epithelial function is closely associated with the resolution of pulmonary edema and better clinical outcomes (Ware and Matthay, 2001). The keratinocyte growth factor (KGF), a paracrine-acting epithelial mitogen, has been validated as an important modulation factor in epithelial repair and is often stimulated by many proinflammatory cytokines in ARDS. KGF may enhance the epithelial barrier integrity in multiple ways, such as being antiapoptotic and cytoprotective, stimulating epithelial cell proliferation, and removing the adverse effect of reactive oxygen species (ROS) (Finch and Rubin, 2004).

Beneficial effects of KGF have been validated in various animal models, including hyperoxia-induced, acid-induced, ventilator-induced, post-allogeneic bone marrow transplant lung injury, and several kinds of pulmonary inflammation models (Ware and Matthay, 2002). As for human studies, Fang et al. developed a reproducible endotoxin-induced ALI model in an *ex vivo* human lung and found that the therapeutic effect of allogeneic mesenchymal stem cells was partly induced by the secretion of KGF through restoring sodium-dependent alveolar fluid transport (Fang et al., 2010). In a pre-clinical human trial, volunteers were randomized to intravenous KGF (60 mg/kg) or placebo for 3 days before inhaling 50 mg LPS. The results showed that KGF treatment increases bronchoalveolar lavage fluid (BALF) surfactant protein D, interleukin-1 receptor antagonist (IL-1Ra), matrix metalloproteinase- (MMP-) 9, and granulocyte-macrophage colony-stimulating factor (GM-CSF) level, indicating its role in epithelial repairment and anti-inflammation regulation (Shyamsundar et al., 2014). However, in one double-blind, randomized, placebo-controlled trial in patients with ARDS, the KGF group (palifermin 60 μg/kg) showed no positive influence on median ventilator-free days, with higher mortality at 28 days and frequent adverse events (Mcauley et al., 2017). The inconsistent result between the pre-clinical and clinical studies may be caused by the lack of KGF receptors on the injured epithelial cell surface or different routes of administration, which require further study to demonstrate.

#### Anti-platelet therapy

Platelets are widely known as critical components of the blood system that regulate hemostasis and can contribute to circulating thrombosis under pathologic conditions. However, recent observations have revealed their properties in vascular endothelial integrity, tissue repairment, and inflammation modulation. Infectious pneumonia, sepsis, aspiration, and trauma are four major high-risk conditions for ARDS, whereas platelets contribute to each of these principle etiologies (Middleton et al., 2018).

In acid-induced, ventilation-induced, and transfusion-related lung injuries, platelets have been validated to be associated with lung neutrophil accumulation, leading to the alveolar-capillary permeability change and pulmonary edema formation (Grommes et al., 2012; Ortiz-Muñoz et al., 2014; Rossaint et al., 2014). Zarbock et al. found that pretreatment of acetylsalicylic acid (ASA), the non-selective cyclooxygenase with antiplatelet effects, blocked the thromboxane- (TX-) A2, which is generated by platelet and involved in the development of ALI, could improve gas exchange, and reduced neutrophil recruitment and permeability by inhibiting the platelet-neutrophil aggregation (Singbartl and Ley, 2007). Kebir et al. demonstrated that ASA-triggered 15-epi-LXA4 (ATL) and its metabolically stable analog ATL promoted the apoptosis of human neutrophils and facilitated the resolution of pulmonary inflammation by overriding the effect of myeloperoxidase (MPO) (Kebir et al., 2009). In the hypertoxic-induced lung injury model, aspirin-triggered resolvin D1 (AT-RvD1) treatment effectively reduced the wet/dry (W/D) ratio of the lung and protein in BALF by disrupting the inflammatory cascade and reducing oxidative stress (Cox et al., 2015).

Recently, one pre-clinical human study showed that aspirin reduced pulmonary neutrophils and MMP-8/MMP-9, TNF-α, TX-B2, and histological damage (Hamid et al., 2017). Nevertheless, in one multicenter, double-blind, placebo-controlled, randomized clinical trial among 390 analyzed patients, the administration of aspirin neither usefully reduced the incidence of ARDS at 7 days nor improved other clinical outcomes, including ventilator-free to day 28 and the length of ICU or hospital stay (Kor et al., 2016). Middleton et al. supposed that platelets show barrier protective and reparative potential in the injured location in the ARDS alveolar, whereas exogenous anti-platelets therapy may interfere with this effect, thus leading to negative clinical outcomes (Middleton et al., 2018). Therefore, a better understanding of the role of platelets in lung biology and its relationship with lung inflammation and injuries is critical for establishing promising therapy for ARDS.

#### Statin

Statins, the 3-hydroxy-3-methylglutaryl coenzyme A (HMG-CoA) reductase inhibitors, can reduce the serum cholesterol level, which underly its therapeutic effect in cardiovascular disease and dyslipidemia. Recent studies demonstrated statin pleiotropic effects such as antioxidant, anti-inflammatory, and endothelial-protective properties (Ito et al., 2006), which favors its potential use in the treatment of ARDS.

Jacobson et al. found that simvastatin could reduce the inflammatory index (MPO activity and total neutrophil count) and modulate the inflammation-associated gene expression of LPS-stimulated mice (Jacobson et al., 2005). Shyamsundar et al. then investigated simvastatin’s anti-inflammatory effect in the LPS-induced ALI healthy volunteers’ model and found it reduced the level of BALF neutrophilia, MPO, MMPs, C-reactive protein (CRP), and plasma CRP (Shyamsundar et al., 2009). McAuley et al. finished a multicenter, double-blind, and randomized clinical trial that demonstrated that the simvastatin therapy had no beneficial effect on multiple clinical outcomes, such as ventilator-free days, days free of non-pulmonary organ failure, and mortality at 28 days (Mcauley et al., 2014a). In contrast, a similar result was presented in a multicenter double-blinded clinical trial about Rosuvastatin therapy in sepsis-associated ARDS (Truwit et al., 2014). This conflict outcome between pre-clinic and clinical trials is probably due to altered drug pharmacokinetics in critically ill patients, which result in inadequate concentrations when given the standard regimens.

In addition, several COVID-19 preclinical studies have shown that statins reduce the incidence of pulmonary vascular events by alleviating platelet aggregation (Undas et al., 2005), venous thrombus formation (Kunutsor et al., 2017), and interfering with viral entry (Undas et al., 2005). It has also shown promising efficacy in reducing COVID-19 mortality, complications, and the number of hospital days based on several current retrospective analyses (Gupta et al., 2021; Lee et al., 2021; Mazloomzadeh et al., 2021; Investigators, 2022). These results suggest that statins may not be appropriate in patients with classical ARDS but with more severe inflammatory such as COVID-19 ARDS.

#### Neuromuscular blocking agents

Ventilator-induced lung injury (VILI) is one of the main causes of death during the management of ARDS (Slutsky and Ranieri, 2013). Pharmacological treatments applying neuromuscular blocking agents (NMBA), which can cause skeletal muscle relaxation by blocking the acetylcholine receptor neuromuscular junction, seem to be a possible solution for VILI (Naguib et al., 2002). In mechanically ventilated patients with ARDS, NMBA can arrest patients against the ventilator to increase its synchrony with the ventilator, minimize respiratory muscle oxygen consumption (Hraiech et al., 2015), and reduce alveolar fluid accumulation (Slutsky, 2010).

The effect of NMBA infusion on gas exchange in ARDS patients has been tested in a relatively small-sized clinical trial. Its results demonstrated that compared with the control group, the PaO2/FiO2 ratio of the test group shows significant improvement (Gainnier et al., 2004). To further validate the safety and efficacy of NMBA therapy, Papazian et al. performed a multicenter, double-blinded trial among 340 early and severe ARDS patients. They found that NMBA early administration improved the 90-day survival and ventilator-free days without increasing the risk of muscle weakness (Papazian et al., 2010).

Besides the respiratory physiology effect, a recent study proved that NMBA also plays an anti-inflammatory role by reducing several pro-inflammatory cytokines (IL-1β, IL-6, and IL-8), which may favor its therapeutic effect (Forel et al., 2006). This anti-inflammation effect has been further studied in the rodent ALI model and several cell lines, which was mediated by blockade of the nicotinic acetylcholine receptor α1 (α1nAChR) (Fanelli et al., 2016). Similar benefits have been validated in the treatment of COVID-19 ARDS (Zareifopoulos et al., 2020; Cook et al., 2021).

### Cytokine-based therapy

#### Interferon-β

Interferons (IFN) are a family of glycoproteins released by host cells in response to pathogens, functioning as antiviral, inhibiting cell proliferation, and regulating immunity (Vilcek, 2006). IFN-β is the most studied concerning ARDS treatment, and its therapeutic effect and mechanism depend on regulating ecto-5′-nucleotidase (CD73), a membrane-bound glycoprotein expressed on vascular endothelium, epithelial cells, and leucocyte (Colgan et al., 2006). In the LPS-induced lung injury model, pulmonary CD73 expression was significantly increased, whereas the depletion of CD73 led to pulmonary neutrophil accumulation and alveolar-capillary leakage (Reutershan et al., 2009). Upregulation of CD73 with IFN-β has shown the effect of preventing vascular leakage and inhibiting leukocyte recruitment (Colgan et al., 2006; Kiss et al., 2007).

Geoff et al. first reconfirmed that IFN-β-1a upregulated the CD73 level in human lungs and then confirmed its safety, tolerability, and efficacy in reducing the 28-day mortality in a multicenter open-label study (Geoff et al., 2014). One randomized, double-blind, placebo-controlled phase 2 trial affirms that inhaled IFN-β-1a (SNG001) in the treatment of COVID-19 significantly reduces patient mortality (Monk et al., 2021). IFN-β also shows better outcomes in patients with COVID-19 ARDS (George et al., 2020) based on its effect of blocking viral replication and promoting humoral and cellular immune responses (Lokugamage et al., 2020).

#### Anti-TNF-α therapy

TNF-α is a key proinflammatory cytokine that takes part in multiple pulmonary inflammatory pathologies *via* various mechanisms, including recruiting leukocytes, promoting leukocyte migration, proliferation, and differentiation, which also participate in the processes of oxidative stress, necrosis, apoptosis, and tissue remodeling (Mukhopadhyay et al., 2006). Among patients with ARDS, TNF-α is elevated in both plasma and BALF (Hyers et al., 1991; Roten et al., 1991) and is strongly associated with worse clinical outcomes (Makabe et al., 2012).

Mullen et al. first demonstrated that pretreatment with combined ibuprofen and TNF-α monoclonal antibody attenuated the course of sepsis-induced ALI in pigs (Mullen et al., 1993). In the rat model of hyperoxia-induced ALI, subcutaneous pretreatment p75 TNF receptor- (TNFR-)Ⅱ human immunoglobulin G1 (IgG1) construct protected the lung from alveolar-capillary leakage and granulocyte accumulation (Guthmann et al., 2009). In contrast, selective application of p55 TNF receptor- (TNFR-)Ⅰ specific domain antibody could inhibit lung injury, edema, and inflammation (Bertok et al., 2011). Recently, scientists developed a novel selective TNFR-Ⅰ antagonist (GSK1995057), which effectively suppressed cytokines and neutrophil adhesion molecule expression in activated human pulmonary microvascular endothelial cells. Moreover, in healthy volunteers, it attenuated inflammation and endothelial injury (Proudfoot et al., 2018).

Cytokine storm is considered one of the main pathogenic factors of COVID-19 ARDS. The massive release of TNF-α is the main characteristic of cytokine storm, suggesting that inhibiting TNF-α may block this excessive inflammatory response (Sinha et al., 2020). This assumption was subsequently affirmed by a case report, showing that the application of TNF-α monoclonal antibodies not only reduced cytokine release but also suppressed vascular endothelial growth factor production, ultimately alleviating the COVID-19 ARDS (Jamilloux et al., 2020).

#### Cytokine antibody

In ARDS pathophysiology, circulating concentrations of IL-1β, IL-6, IL-8, TNF, and other cytokines are known to cause an increased pro-inflammatory effect. During the COVID-19 pandemic, ARDS has become a deep understanding of the concept of cytokine balance. In order to counteract the accumulation of cytokines, cytokine antibody has been developed. Part of the research, such as the microfluidic single-cell study, illustrated the protection role of IL-1β, IL-6, or TNF-like cytokine antibodies in early ARDS (Preira et al., 2016). IL-8 is another endogenous chemotactic factor for neutrophils. Its antibody is also used to reduce the severity of ARDS. Bao et al.(2010) found that the humanized anti-IL8 antibody prevented neutrophil infiltration and alleviated the ARDS syndrome.

In clinical research, such as the RECOVERY trial (Lewis, 2021) and the REMAP-CAP study (Rosas et al., 2021), tocilizumab showed its benefits in COVID-19 ARDS treatment as an IL-6 receptor monoclonal antibody combined with high-dose dexamethasone. Meanwhile, canakinumab, a monoclonal antibody targeting IL-1β, showed its effects on ARDS treatment. One clinical case report (Caracciolo et al., 2020) demonstrated that applying canakinumab prolonged the life duration of a high-risk, old ARDS patient and prevented them from multiorgan damage due to COVID-19. However, Mastroianni et al. (2021) conducted a retrospective, observational analysis of using canakinumab (300 mg, s.c.) in the early stage of COVID-19. The data show that this treatment reduced the need for MV, and the serum pro-inflammatory markers also decreased without adverse events.

### Inhalation agents

Inhalation therapy using the aerosol delivery system is a promising alternative due to its unique properties. By nebulizing, it can deliver medication directly to the target region and cooperate with the respiratory movement. Moreover, the inhaled agents reach the circulation rapidly through immense absorption and diffusion capabilities of the lung, avoiding the relatively low local efficacy and high risk of systematic side effects by intravenous administration.

#### β-Agonists

Beta-adrenergic agonists are commonly used as bronchodilators in treating patients with airway obstruction. In recent decades, numerous studies have revealed its potential use in ARDS by increasing intracellular cyclic adenosine monophosphate (cAMP) levels. Beta-adrenergic agonists can activate sodium and chloride trafficking across the alveolar epithelium by stimulating the β-2 receptor to upregulate cAMP, which re-creates the osmotic gradient for water reabsorption from the damaged ARDS alveoli (Berthiaume and Matthay, 2007). It also stimulates cellular repair and inhibits NF-кB by activating the protein kinase A (PKA) pathway (Farmer and Pugin, 2000; Perkins et al., 2008).

BALT I, a single-center, phase 2 randomized controlled trial (RCT), first validated a significant reduction of the extravascular lung water with salbutamol infusion for 7 days (Perkins et al., 2006). However, subsequent trials fail to present consistent efficacy. BALT II, another randomized, double-blind, placebo-controlled, multicenter trial conducted by BALT I investigators, was stopped by the Data Monitoring and Ethics Committee (DMEC) because of the adverse effect of salbutamol on 28-day mortality (Gao et al., 2012). Likewise, the aerosolized β-agonists for treatment of acute lung injury (Drug Study of Albuterol to Treat Acute Lung Injury, ALTA) trial were stopped early due to undesirable clinical outcomes such as ventilator-free days and mortality (Matthay et al., 2011). The researchers also found that perioperative inhaled salmeterol negatively affects on the incidence of ALI among patients undergoing elective esophagectomy (Perkins et al., 2014).

The inconsistency between basic study and clinical practice may be explained by several underlying mechanisms: 1) epithelial injury during ARDS can limit the interaction between β-agonists and its receptor, whereas continuous administration of β-agonists downregulates the expression of receptors, thus leading to unresponsiveness; 2) β-agonists have a positive inotropic effect on the myocardium, increasing cardiac output and exacerbating alveolar-capillary leakage; and 3) β-agonists could stimulate the RAAS, which could hinder the effect of AFC (Thompson, 2012). Because it lacks the benefit and suggests the tendency to cause harm, such as arrhythmia and tachycardia, β-agonists may not be considered a routine solution.

#### Heparin

The excessive activation of immune and clotting systems during ARDS leads to fibrin deposition around the pulmonary microcirculation and alveolar hyaline membrane formation, further promoting lung dysfunction and accelerating pulmonary fibrosis (Idell, 2003). On the one hand, heparin, a glycosaminoglycan, reinforces the anti-coagulate capability of antithrombin (AT) to exert its anti-coagulant effect (Young, 2008). On the other hand, it inhibits the activation of NF-κB and reduces the production of inflammatory mediators to exert its anti-inflammation effect (Li et al., 2013).

A phase I trial that tests various dosages of nebulized heparin among mechanically ventilated patients with ARDS found that nebulized heparin was hardly associated with serious adverse events, whereas administrating ≥400,000 U/day showed a reduction in pulmonary coagulopathy (Dixon et al., 2008). Two other randomized controlled trials demonstrated that although the administration of nebulized heparin showed no improvement in arterial oxygenation or mortality, it partly reduced the atelectasis, improved CO2 elimination rate, and increased the ventilator-free day (Dixon et al., 2010; Dixon et al., 2016).

Studies have found the incidence of thrombotic events to be as high as 35% in the more severe COVID-19, which is strongly associated with poor prognosis (Medina-Gómez, 2021). A retrospective analysis including 4,297 mild-to-moderate COVID-19 patients showed that 84.4% of patients were anticoagulated with heparin (subcutaneous injection). Compared to patients who were not anticoagulated, heparin treatment effectively reduced the 30-day mortality (Rentsch et al., 2021). Further evaluation of the efficacy of nebulized heparin therapy is currently ongoing (Van Haren et al., 2020).

#### Surfactant

Pulmonary surfactant is a phospholipoprotein produced by type II alveolar cells, which can lower the alveolar surface tension and maintain the small airway functional integrity (Frerking et al., 2001). Surfactant deficiency and inactivation are prominent mechanisms of the exudative phase of ARDS and lead to alveolar collapse and edema (Günther et al., 2001).

In one of the early studies conducted by Willson et al. involving 42 patients, the oxygenation, earlier extubation, and intensive care requirement were ameliorated by the administration of calf lung surfactant extract (calfactant). However, the overall mortality shows no obvious difference between treatment and control groups (Willson et al., 1999). The same research team further conducted another large multicenter, randomized clinical trial involving 152 patients, demonstrating that the calfactant administration improved oxygenation and reduced mortality but hardly affected the duration of ventilation therapy or hospital stays (Willson et al., 2005a). The *post hoc* analysis of this trial demonstrated that exogenous surfactant therapy was only effective among patients with direct lung injury such as pneumonia, aspiration, and drowning. Hence, the investigators further conducted a trial focused on this subgroup. However, results from this group showed that surfactant administration not only brought no beneficial effect but also may prolong the duration of ventilation and increased the need for critical care, ultimately illustrating the unsuccessful use of exogenous surfactants to treat typical ARDS (Dushianthan et al., 2012; Willson et al., 2013).

Thus, despite its potential in treating infants and children ARDS patients, exogenous surfactant therapy failed to demonstrate positive results in several clinical trials and may associate with adverse effects such as hypoxia and hypotension (Willson et al., 2005b; Davidson et al., 2006). The following study may need to optimize delivery strategy and ascertain targeted patient subgroups (Kim and Won, 2018).

#### Nitric oxide

Nitric oxide (NO) can stimulate the formation of cyclic guanosine 3′,5′-monophosphate (cGMP) and further suppress the calcium-induced contraction either by decreasing the myosin sensitivity or lowering the intracellular calcium concentration (Griffiths and Evans, 2005). When administrated by inhalation, NO could pass through the alveolar-capillary barrier and selectively mediate the smooth muscle relaxation and pulmonary vascular dilation, thus being beneficial to the ventilation/perfusion mismatch during ARDS.

Rossaint et al. conducted the first study among ARDS patients. They found that inhalation NO (iNO) could reduce pulmonary arterial pressure and increase oxygenation (Rossaint et al., 1993). According to several subsequent clinical trials or analyses, although the iNO therapy may improve short-term oxygenation, it presents little effect on mortality and ventilation-free days (Michael et al., 1998; Taylor et al., 2004; Adhikari et al., 2013). Therefore, up-to-date, routine application of iNO may not be recommended due to its high risk of causing hypoxemia or hypoxia (Germann et al., 2005). However, considering reopening the atelectatic alveolar could favor the selective vasodilatation of iNO, it is reasonable to combine pulmonary recruitment maneuvers (positive end-expiratory pressure (Johannigman et al., 2000), prone position ventilation (Germann et al., 1998), and high-frequency oscillation (Mehta et al., 2003)) with iNO to improve the gas exchange. A few cases and *in vitro* studies have shown the underlying therapy effect of NO in COVID-19 ARDS (Chen et al., 2004; Zamanian et al., 2020), but its utility still requires a more robust clinical trial to prove.

### Corticosteroid

Corticosteroids are steroids produced by the adrenal cortex, including glucocorticosteroids, salt corticosteroids, and sex hormones. Given its broad immunomodulation properties, such as suppressing the pro-inflammatory cytokines, inhibiting neutrophil activation, and promoting the efficacy of anti-inflammatory molecules, corticosteroids have long been considered the potential therapy of dysregulated inflammation for ARDS (Hooper, 1991; Cronstein et al., 1992).

Bernard et al. found that applying methylprednisolone (30 mg/kg/6 h) had no effect on clinical outcomes among patients with established ARDS (Bernard et al., 1987). These results were consistent with several other trials that showed that high-dose, short-course corticosteroids failed to improve the mortality of early-phase ARDS (Sprung et al., 1984; Luce et al., 1988). However, according to a *post hoc* analysis, 7-day treatment with a low dose of corticosteroids improved survival and reduced ventilation days over the course of 28-day (Annane et al., 2006). A subsequent clinical trial evaluating the safety and efficacy found that a moderate dose of methylprednisolone administration attenuated organ dysfunction and reduced the duration of MV and ICU stay length (Meduri et al., 2007).

In the corticosteroid treatment of COVID-19 ARDS, low-dose dexamethasone (DEX, 6 mg once daily for 10 days) given orally or intravenously to patients receiving invasive MV reduced the 28-day mortality (Group, 2021). A retrospective cohort study showed that treatment with methylprednisolone also reduced mortality from COVID-19 ARDS (Wu et al., 2020). Nevertheless, after the onset of ARDS, corticosteroid administration for at least 14 days was closely related to increased 60- and 180-day mortality rates despite the ventilator-free and shock-free days improved among the first 28 days (Steinberg et al., 2006). This may partly be explained by the hypothesis that the initial benefit of corticosteroid therapy in ARDS works through suppressing the inflammatory process and reducing alveolo-capillary leakage. However, it soon offsets due to delayed adverse effects such as immunosuppression, tissue repair disorder, and delayed viral clearance (Ruan et al., 2014; Aran et al., 2018). Thus, corticosteroid therapy is not recommended for the routine management of ARDS and needs further study about its administration time window and long-term effects.

### Cell-based therapy

The injury mechanisms of ARDS are quite complex and diverse, and the severity and characteristics of patients are quite heterogeneous. This indicates that using one single target mediator or pathway is inadequate to achieve a therapeutic effect and may explain the failure of multiple pharmacotherapies for ARDS clinical trials (Tonelli et al., 2014). Cell-based therapy serves as a rising approach for ARDS treatment due to its multiple functions, including modulating immune response (Curley et al., 2012; Curley et al., 2013; Devaney et al., 2015), enhancing bacterial clearance (Krasnodembskaya et al., 2012; Lee et al., 2013), improving inflammation resolution (Curley et al., 2012; Curley et al., 2013), and restoring capillary barrier function (Fang et al., 2010; Goolaerts et al., 2014), whereas mesenchymal stem/stromal cells (MSC) have unique advantages over the others based on their good tolerance and simple preparation.

This therapy has been tested in many clinically relevant models of ARDS and proved its translation potential. Devaney et al. demonstrated that human bone marrow-derived MSC reduced bacterial load, suppressed inflammation, decreased lung injuries, and improved survival in a rodent model of ALI induced by *E. coli* infection (Devaney et al., 2015). This therapeutic effect was also well-validated in a live bacteria-induced *ex vivo* human lung injury model (Lin et al., 2013). In addition to bacterial infection, MSCs have proved their therapeutic potential in treating H9N2 avian influenza virus-induced ALI (Yan et al., 2016) and attenuating the H5N1 lung injury in mice (Chan et al., 2016). McAuley et al. found that MSCs can enhance the absorption of pulmonary edema in an *ex vivo* preparation of perfused human lungs (McAuley et al., 2014a). The clinical trial has proved the well-toleration of the intravenous administration of human bone-marrow among patients with moderate-to-severe ARDS (Wilson et al., 2015). Researchers designed a phase 2a trial with the primary focus on safety, which may be followed by a larger 2b trial with the efficacy endpoints. As for the use of MSC in COVID-19 ARDS, studies show that MSC treatment can attenuate cytokine storm associated with COVID-19 ARDS infection (Mehta, McAuley, et al., 2020). In contrast, a phase I clinical study already affirmed the safety of MSC in treating COVID-19 ARDS (Enes and Weiss, 2020).

Therefore, to further advance the therapeutic potential of MSC in typical or COVID-19 ARDS, more studies may be needed to focus on various aspects, including reducing cell batch heterogeneity, optimizing the dosage and administration route, and targeting the suitable patient population.

### Natural medicine therapy

Since ancient times, natural herbs have been used in treating inflammation and other disorders, especially in China. Over the past thousand years, natural herb medicines have proved their unique advantages, such as low adverse effects, multiple targets, and plentiful resources, so traditional wisdom may provide us with some innovations in treating ARDS.

#### Honokiol

In Traditional Chinese Medicine, the magnolia bark extract (MBE) has been widely used alone or combined with other herbal-derived compounds in various pathological conditions for its sedative, antioxidant, anti-inflammatory, antibiotic, and antispastic properties (Sarrica et al., 2018). Honokiol is a small molecule MBE with a molecular formula of C_18_H_18_O_2_ that has been shown to exhibit antiangiogenic, anti-inflammatory, and antitumor properties (Fried and Arbiser, 2009; Lee et al., 2011).

In the animal model of sepsis-associated ALI, instead of exerting a direct anti-microbial effect, honokiol inhibited the function of multiple pro-inflammatory cytokines such as TNF-α and high mobility group box-1 protein (HMGB1), suppressing the production of inducible NO synthase- (iNOS-) mediated NO and the activation of NF-κB, ultimately improving the outcome of ALI (Weng et al., 2011). However, in the LPS-induced ALI animal models, honokiol could activate the sirtuin 3 (SIRT3)/AMP-activated protein kinase (AMPK) axis and inhibit the expression of Ang-Ⅱ to protect the pulmonary microvascular endothelial barrier (Lan et al., 2018). Moreover, honokiol could attenuate lung injury by inhibiting NOD-like receptor protein 3 (NLRP3) mediated pyroptosis *via* the activation of NF-E2-related factor 2 (Nrf2) (Liu S et al., 2021).

#### Isoforskolin

*Coleus forskohlii* belongs to the Lamiaceae family, which is widely distributed in Yunnan (China), India, and Thailand and has been documented to contain several bioactive compounds. Isoforskolin (ISOF) was isolated from *Coleus forskohlii* and has been validated to be able to activate adenylyl cyclase (AC) and its isoforms, which led to the increased cyclic adenosine monophosphate (cAMP) level (Pinto et al., 2008). Several studies have demonstrated that cAMP supplement therapy was associated with better outcomes of ALI (Sciuto et al., 1996; Takeoka et al., 1997).

Based on the above findings, Yang et al. investigated whether the pretreatment of ISOF could ameliorate LPS-induced ALI, and they found the therapy could improve the epithelial and endothelial integrity, promote the clearance of edema, and suppress the level of pro-inflammatory cytokines in several models, including in situ perfused rat lungs and rodent endotoxic shock, and the underlying mechanism may be the interactions between ISOF and PMN mediated by cAMP (Yang et al., 2011). Other mechanisms by which ISOF attenuates lung inflammation have been validated in a chronic obstructive pulmonary disease model, including activation of adenylyl cyclase and downregulation of T helper cell 17 (Th17)/interleukin-17A (IL-17A) and NF-κB/NLRP3 (Xiao et al., 2021a; Xiao et al., 2021b).

#### Caffeic acid phenethyl ester

Propolis of honeybee hives has been listed as one of the major ingredients of traditional diet therapy due to its multiple beneficial properties for centuries (Akyol et al., 2015). Caffeic acid phenethyl ester (CAPE), one of the components extracted from honeybee propolis, has been confirmed to exhibit anti-inflammation and antioxidant effects, which favored its potential therapeutic use in ARDS.

Several studies have tested this hypothesis, and in the LPS-induced rat ALI models, CAPE showed a therapeutic effect in relieving inflammation and lung tissue damage (Koksel et al., 2006). In the subsequent research, Fidan et al. found that CAPE therapy could not only decrease the levels of inflammatory levels but also reduce mortality in sepsis-associated lung injury (Fidan et al., 2007). Based on these studies, Kim et al. tried to uncover the underlying regulatory mechanism and showed that CAPE could interrupt LPS binding to myeloid differentiation protein 2 (MD2) (So et al., 2013). By directly binding to LPS, MD2 induces the formation of the LPS-MD2- TLR4 complex and leads to the activation of the downstream pro-inflammatory signal. CAPE could impair the interactions between LPS and MD2/TLR4 and exert its immunomodulation function. Recently, Chen et al. discovered a novel CAPE derivative with a remarkable protective effect against LPS-induced ALI (Chen et al., 2018). These above mentioned studies provide the idea of developing an efficientMD2 inhibitor for treating ARDS.

#### *Callicarpa japonica* Thunb

*Callicarpa Japonica* Thunb. (CJT) is a tree with purple berry fruits and is widely distributed in the warm seashore area of China and Korea. Nowadays, it is usually cultivated as an ornamental plant. However, its leaves have been used as an herbal treatment. CJT and its metabolites have been considered to possess anti-oxidation and anti-inflammation properties, which could be helpful for ARDS patients.

Shin et al. found that pretreatment with CJT (30 mg/kg) in the mice with LPS-induced ALI decreased the number of inflammatory cells and cytokines and inhibited the inflammatory cell infiltration in the pulmonary tissue (Shin et al., 2015). The anti-inflammation function of CJT may largely depend on its active components, active glycosides and forsythoside B. Active glycosides exert their anti-inflammatory effect in human umbilical vein endothelial cell (HUVECS) by inhibiting cell adhesion molecular (CAM) through suppressing extracellular regulated protein kinases (ERK) and c-Jun NH2 terminal kinase (JNK) phosphorylation (Chen et al., 2009). Jing et al. further investigated the therapeutic effect of active glycosides on the mice LPS-induced ALI model. They concluded that the pretreatment of active glycosides inhibited inflammatory cell infiltration and inflammatory cytokine expression, improving the clinical outcomes *via* inhibiting the NF-κB pathway (Jing et al., 2015). As for forsythoside B, investigators have found it could inhibit inflammation and improve the outcomes of sepsis through modulation of the IκB kinase (IKK) pathway (Jiang et al., 2012).

#### Salidroside

*Rhodiola rosea* is widely known by the common name Roseroot or golden root and has a long history of serving as a natural remedy in ameliorating fatigue and improving cognition and physical conditions. Salidroside is one of the bioactive components of *Rhodiola rosea* and has been reported to possess various pharmacological properties, such as antioxidative and anti-inflammatory effects (Kosakowska et al., 2018). Multiple types of research have explored its potential application in the treatment of ARDS.

Guan et al. first demonstrated in the LPS-induced ALI model that pretreatment of salidroside inhibited the inflammatory cytokines (TNF-α, IL-6, and IL-1β), relieved pulmonary edema, and reduced the number of inflammatory cells in the BALF (Guan et al., 2012). This theory was reconfirmed by Jing et al., and the protective effect of salidroside in the LPS-induced ALI may be through inhibiting the caveolin-1 and TLRs/NF-κB pathway (Jingyan et al., 2017). Salidroside has also proved its beneficial effect in the paraquat-induced rat ALI model (Zhang et al., 2014; Zhang Z et al., 2014). The research by et al. implied that salidroside alleviated ventilation-induced lung injury and the underlying mechanism was probably through inhibiting the activation of NLRP3 inflammasomes by regulating the silent information regulator 1 (SIRT1) function (Wang et al., 2017). A similar mechanism was also discovered in the sepsis-induced ALI model. In the early phase, salidroside inhibited the pro-inflammatory NF-κB pathway through regulating SIRT1, and in the late phase of sepsis, its beneficial effect was approximately through modulating SIRT1-mediated HMGB1 nucleocytoplasmic translocation (Lan et al., 2017).

#### Xuebijing

Xuebijing injection (XBJ) is a Chinese patent drug consisting of *Carthamus tinctorius*, Paeoniae Radix, *Salvia divinorum*, *Angelica sinensis*, and *Ligusticum wallichii* Franch. According to a case report in 2009, XBJ has proved its great potential as a novel option for managing Paraquat poisoning (Sun et al., 2009). In the following years, XBJ has been studied in various medical fields.

As for ALI, XBJ and its compounds upregulated Toll-interacting protein (Tollip) (Liu M et al., 2014) and inhibited the activation of TLR4 and mitogen active protein kinases pathway (Liu Y et al., 2014), thus restraining the downstream pro-inflammatory molecules expression and upregulating the protective IL-10 level (Wang et al., 2014). Furthermore, XBJ has a protective effect on ALI induced by left ventricular ischemia/reperfusion (I/R) in rabbits. This may be through regulation of the inflammatory mediator TNF-α and intercellular adhesion molecule 1 (ICAM-1) expression (Ji et al., 2016). According to a meta-analysis that enrolled 16 randomized controlled trials, XBJ may improve the 28-day mortality rate and symptoms in critically ill patients without severe adverse effects (Li et al., 2018). However, the quality of current evidence is quite low. Therefore, multicenter, precision-designed, randomly assigned trials are needed to evaluate the clinical value.

#### Eriodictyol

Eriodictyol is a bioactivation flavonoid extracted from the traditional Chinese herb *Dracocephalum rupestre*. It has been shown to inhibit LPS-induced pro-inflammatory cytokine production by macrophages through the blockage of NF-κB and phosphorylation of MAPK (Lee, 2011). This is in favor of its potential application in the treatment of ARDS.

In the LPS-induced lung injury model, eriodictyol activated the Nrf2 pathway and reduced the level of cytokines produced by macrophages, thus alleviating lung tissue damage and prolonging the survival of the experimental animals (Zhu et al., 2015). Wang L et al. (2020) and Wang M et al. (2020) found that eriodictyol could attenuate inflammatory mediators in BALF of mice, including IL-6, IL-1β, prostaglandin E2 (PGE2), and TNF-α, and could increase the activity of superoxide dismutase. These may be related to its regulation of the involved cyclooxygenase-2 (COX-2)/NLRP3/NF-κB signaling pathways. More animals should be included and more clinical trials should be conducted to explore its role and mechanism in treating ALI/ARDS.

#### Asperuloside

Asperuloside (ASP) is a component extract from the traditional Chinese herbal medicine Herba Paederiae. It is an iridoid with a molecular formula of C_18_H_22_O_11_ and can inhibit NF-κB and MAPK signaling pathway activation and iNOS expression (Qiu J et al., 2016; He et al., 2018). ASP has been confirmed to have anti-inflammatory, antioxidant, and immunomodulatory effects, which provide a potential basis for its use in treating ALI/ARDS.

Recently, mouse monocyte/macrophage-like cells (RAW 264.7) were pretreated with ASP for 1 h, followed by LPS treatment for an additional 24 h, and it was found that ASP could significantly inhibit the activation of the NF-κB/AMPK signaling pathway (Ke et al., 2018). Similarly, Qiu et al. demonstrated that ASP had a protective effect on LPS-induced RAW 264.7 macrophage by downregulating the inflammatory mediators such as TNF-α, IL-1β, and IL-6, so they investigated its potential therapeutic effect in mice LPS-induced lung injury model and found it attenuated lung edema and lung injuries partly through inactivating NF-κB and MAPK signaling pathways (Qiu Y et al., 2016). Nevertheless, whether ASP can directly inhibit the upstream proteins of the NF-κB has not been elucidated. Therefore, the anti-inflammatory molecular mechanism of ASP is the focus of future research.

#### Paeoniflorin

Radix Paeoniae Alba, commonly known by Bái Sháo, is a herbal medicine applied in Traditional Chinese Medicine for thousands of years. Paeoniflorin (PF) is the main active ingredient of Radix Paeoniae Alba. It reduces the level of intracellular calcium ions, suppresses the expression of proinflammatory cytokines, and has a dual effect on the proliferation and differentiation of lymphocytes, which indicates its anti-inflammation function (He and Dai, 2011). Therefore, they have been used to treat ALI/ARDS.

Kim and Ha showed that PF reduced the levels of NO and PGE2 and protected RAW 264.7 macrophages against LPS-induced injury (Kim and Ha, 2009). Cao et al. further investigated the potential therapeutic effect of PF in the LPS-induced ALI model. They found that PF pretreatment reduced the TNF-α and IL-1β levels while promoting the production of IL-10. The mortality and multiorgan function significantly improved compared with the control group (Cao et al., 2011). Zhou et al. found that PF could significantly downregulate the expression of IL-1β and TNF-α and inhibit the phosphorylation of MAPK and JNK signaling and the activation of NF-κB, thereby attenuating LPS-induced ALI in mice (Zhou et al., 2011). Furthermore, in an influenza A virus- (IAV-) induced ALI model, Yu et al. found that PF could improve the survival rate of infected mice and reduce viral titers and pulmonary fibrosis in lung tissue, and the mechanism may be related to the reduction of the virus titer in lung tissue by downregulation αvβ3 (a type of integrin)/TGF (transforming growth factor)-β 1 pathway activation is associated with reduced proinflammatory cytokine production (Yu et al., 2021). Given the numerous advantages of PF, more trials are warranted for further research.

#### Curcumin

*Curcuma longa* is an herb with good medicinal benefits that have been widely used in Traditional Chinese Medicine for hundreds of years. Curcumin (Cur) is a compound extracted from the rhizome of turmeric with a molecular formula of C_21_H_20_O_6_. Recently, it has been found to possess various pharmacological properties, such as anti-inflammatory, antioxidant, and immunomodulatory properties (Mirzaei et al., 2017).

In the past few years, there have been many preclinical studies on Cur therapy for ALI/ARDS. These studies found that Cur can decrease neutrophils (Bansal and Chhibber, 2010; Suresh et al., 2012; Tyagi et al., 2014; Kumari et al., 2015; Xu et al., 2015; Kim et al., 2016; Qian et al., 2019; Hu et al., 2020; Wang et al., 2021) and macrophages (Kim et al., 2016; Qian et al., 2019; Chai et al., 2020) in BALF to exert anti-inflammatory effects and can also decrease the expression of NO (Tyagi et al., 2014; Kumari et al., 2015) and malondialdehyde (MDA) (Kumari et al., 2015) to exert antioxidant effects. Moreover, Cur can inhibit the expression of inflammatory factors, including TNF-α (Bansal and Chhibber, 2010; Suresh et al., 2012; Tyagi et al., 2014; Kumari et al., 2015; Xu et al., 2015; Kim et al., 2016; Qian et al., 2019; Wang et al., 2021) and IL-6 (Bansal and Chhibber, 2010; Suresh et al., 2012; Kumari et al., 2015; Xu et al., 2015; Kim et al., 2016; Qian et al., 2019; Wang et al., 2021). Moreover, intranasal inhalation administration of Cur has better efficacy than traditional oral administration (Tyagi et al., 2014; Kumari et al., 2015; Hu et al., 2020), which may be because inhalation administration can directly target the lungs and improve the drug concentration and availability. Therefore, it is reasonable to further define its mechanism of action and carry out large clinical studies.

## Nanomedicine

### What is nanomedicine?

Nanoscience is the applied science for studying the methods and composition of materials and devices at the nanoscale (10–1,000 nm). Nanomedicine is the application of nanotechnology to medicine (Bodmeier et al., 1991). It not only takes advantage of nanotechnology but also acts at the cellular level to exert its effects (Prow et al., 2004). In recent years, nanomedicine has been widely used to treat ALI/ARDS.

### Classification of nanomedicine

Nanomedicine refers to using nanofabrication technology to fabricate drugs and compounds, among others, into nanoscale particles or to combine various carrier materials with drugs and compounds to form nanoscale particles or drug formulations. The resulting nanomedicines can not only exhibit nanoscale but also present nanoeffects (Qiao et al., 2021). Nanomedicines mainly include drug nanosizing and drug delivery systems (DDS) with nanomaterials as carriers.

#### Drug nanosizing

As the name implies, drug nanosuspension is the process by which large particulate drugs, mainly poorly soluble, are broken down into small particles and prepared as nanosuspensions *via* methods such as grinding or homogenization (Bodmeier et al., 1991). Many drugs are water-insoluble and, therefore, poorly bioavailable, whereas nanosuspensions can load with a large amount of poorly water-soluble drugs, which greatly improve drug solubility and dissolution rates (Gulati et al., 2021).

#### Nanodrug delivery system

Nano-DDS are pharmaceutical applications of nanotechnology and have shown promising promise in diagnosis and therapy, prolonging drug release time, improving drug solubility, limiting systemic exposure, and reducing toxic side effects of drugs (Koo et al., 2005; Patton and Byron, 2007). DDS with nanomaterials as carriers can target the transport of therapeutic drugs to the lungs to exert therapeutic effects (Dames et al., 2007; Foldvari and Elsabahy, 2011; Sadikot et al., 2017).

**FIGURE 2:**
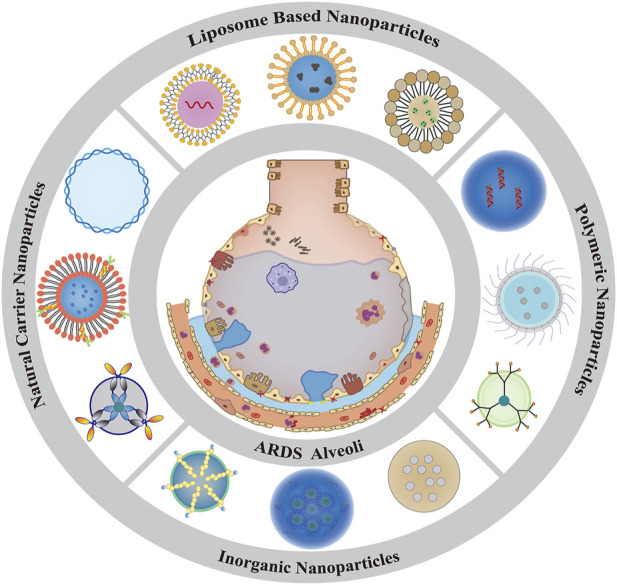
Summary graph of the current common classification of nanodrug delivery system.

Over the past few years, several nanocarrier-based DDS have been developed to specifically deliver drugs in ALI/ARDS (Table 2) using lipids (Lim et al., 2011), organic polymers (Salik et al., 2016), natural nanoparticles (Bohr et al., 2017), and inorganic nanoparticles (Rijt et al., 2016) (Figure 2). These nanocarriers can improve the stability and solubility of drugs in the lung and prolong the circulation and release time to improve the safety and efficacy of drugs (Longfa et al., 2018).

**TABLE 2:** Application of different nanomedicines for the treatment of ALI/ARDS.

Drugs/gene	Materials	Size (nm)	Model	Dose	References
Liposome-based nanoparticles					
TNF-α	Lecithin, chol, a lipid-polyethylene glycol conjugate	370.0 ± 96.9	J774A.1 cell line	48 mg/ml in cells	Wang et al. (2020)
NF-κB	Sugar-C4-Chol derivative, DOTMA, chol	100	Male Wistar rats	5 mg/kg in rats	Wijagkanalan et al. (2011)
Dexamethasone	DSPC	110 ± 6.9	Male Wistar rats	0.5 mg/kg in rats	Wijagkanalan et al. (2008)
Methylprednisolone	DSPC, chol	106	Male SD rats	1, 0.5 mg/kg in rats	Li N et al. (2017)
Sivelestat	DOPC, MPB	266 ± 12	Female BALB/c mice	50 mg/kg in mice	Okeke et al. (2020)
Omeprazole	Egg yolk lecithin, DiD, chol	144.9 ± 1.1	C57BL/6 wild-type male mice, THP-1 cell line	12.6 mg/kg in mice, 5 mg/ml in cells	Sun et al. (2022)
EUK-134	DPPC, DSPE-PEG	197.8 ± 4.5	HUVECs, C57BL/6 male mice	1.5 g/ml in cells, 3.2 mg total lipid at 2000 CPM/μl in mice	Howard et al. (2014)
MiRNA-26	—	71.5	Human small airway epithelial cells, CD-1 mice	0.75 mg/ml in cells, 2 mg/kg in mice	Zhou et al. (2018)
MiRNA-126	—	30–120	Human endothelial progenitor cells, CD-1 outbred mice	40 μM/ml in cells, 70 μg/kg in mice	Zhou et al. (2019)
MiR-28-5p	—	50–200	Male SD rats, rat MSCs	50 μg/ml in cells	Kor et al. (2016)
Polymeric nanoparticles					
Venetoclax	PEG, α-lipoic acid	∼102	Male BALB/c mice, mouse RAW264.7 cell line	10 mg/kg, 40 μM/ml	Su et al. (2021)
DC-SIGN	Phosphorhydrazone, N-BOC-tyramine		Female BALB/c and C57BL/6 mice, human monocyte-derived DCs	1 mg/kg in mice, 5, 10 μg/ml in cells	Blattes et al. (2013)
Curcumin	TBP, C11G3	40.2	MH-S cells, Male Balb/c mice	5–20 μM/ml in cells, 21.83 mg/kg in mice	Li et al. (2022)
	PEG, PLGA	250 ± 9.16, 423 ± 5.57, 851 ± 31.8	HUVECs, ICR male mice	3–200 μg/ml in cells, 1 mg/kg in mice	Jin et al. (2019)
Curcumin, HO-1	PAMG2, PEI25k, cholesteryl chloroformate	120	The L2 cell line, male BALB/c mice	Wt ratio for cells transfection was 1:15 for pDNA/PamChol-Cur, 75 μg/kg in mice	Kim et al. (2019)
HO-1, GA	PAM generation 2, PEI, DIPEA	107.1 ± 28	C6, HEK293, L2, and Raw264.7 cells, BALB/c mice	Wt ratio for cells transfection was 1:9:8 for pDNA/PamHRchol/GA, 8 μg/kg in mice	Choi et al. (2021)
Dexamethasone	PEI, MTC-mannose, PCL	156 ± 1	Male Kunming mice, HUVECs, mouse RAW264.7 cell line	2 mg/kg in mice, 10, 5, 2.5, 0.5 μg/ml in cells	Su et al. (2022)
Aspirin	Poly-A polymer, EDC, NHS	1000	Male Balb/cJ or C57BL/6J mice	≈30 mg/kg in mice	Brannon et al. (2022)
Selenium	SiO_2_	50	Male SD rats and RLE-6TN cells	1 mg/kg in rats, 80 μg/ml in cells	Yong et al. (2017)
Inorganic nanoparticles					
Dexamethasone	SiO_2_	100	CD-1 mice, THP-1, and A549 cell lines	25 mg/kg in mice, 2 μg/ml in cells	García-Fernández et al. (2021)
Curcumin	FeCl_3_, PVP	400	J774A.1 cells, male BALB/c mice	0.1 μg/ml in cells, 8 mg/kg in mice	Yuan et al. (2021)
	GNPs (CLPFFD)	18.8 ± 0.1	Male C57BL/6 mice	500 nM, 50 μl/mouse	Wang X et al. (2020)
CeO_2_	CeO_2_@SiO_2_	220 ± 5	Male Wistar rats	0.6 mg/kg	Serebrovska et al. (2017)
	GNPs (CLPFFD)	23.9 ± 0.3	Male C57BL/6 mice, THP-1 cells	500 nM in mice, 100 nM in cells	Gao et al. (2019a)
	GNPs (CLPFFD)	26.9 ± 0.8	Male C57BL/6 mice, THP-1 cells	500 nM in mice, 100 nM in cells	Gao et al. (2019b)
	Se@SiO2 PVP coated	∼55	Beas-2B cell line, male C57BL/6 mice	10 μg/ml in cells, 100 μg/kg in mice	Wang et al. (2020)
Natural carrier nanoparticles					
ALX-0171			HEp-2 cells, male and female cotton rats	1–16 nM in cells, 100 µl in rats	Detalle et al. (2016)
Methylprednisolone	Chitosan	120.6, 233.3	Male Fischer rats and BALB/C mice	12 mg/kg in rats and mice	Ding et al. (2022)
TPCA-1	DiO, Dil, DiD	200	HUVECs, HL60 cells, adult CD1 mice	450 ng/ml in cells, 1 mg/kg in mice	Gao et al. (2016)
	DiD	100–150	BALB/c mice, RAW264.7 cells, HUVECs	1 mg/kg in mice, 600 mg/ml in cells	Ma et al. (2020)

DOTMA, low-density lipoprotein receptor 1;1-(2,3-dioleyloxy) propyl]-N,N,N-trimethylammonium chloride; DSPC, 1,2-dis-tearoyl-sn-glycero-3-phosphocholine; Chol, cholesterol; SD, Sprague Dawley; DOPC, 1,2-dioleoyl-sn-gly-cero-3-phosphocholine; MPB, 1,2-dioleoyl-sn-glycero-3-phos-phoethanolamine-N-[4-(p-maleimidophenyl)butyramide] sodium salt; DPPC, 1,2-dipalmitoyl-sn-glycero-3-phosphocholine; DC-SIGN, DC-specific intercellular adhesion molecule 3-grabbing nonintegrin; DC, dendritic cells; TBP, tyramine-bearing two dimethyl phosphonate sodium salt; C11G3, amphiphilic phosphorus dendron; TPP, triphenylphosphonium cation; DIPEA, cholesteryl chloroformate, N,N-diisopropylethylamine; GA, glycyrrhizic acid; EDC, N-(3-dimethylaminopropyl)-N′-ethylcarbodiimide hydrochloride; NHS, N-hydroxysuccinimide; PVP, poly(vinylpyrrolidone); CSE, cigarette smoke extract.

##### Liposome-based nanoparticles

Liposomes typically range in size from 50 nm to 1 μm (Allen and Cullis, 2013), with the bilayer mainly composed of phospholipids and cholesterol, both oleophilic and hydrophilic. Therefore, liposomes are the most widely used and the closest to clinical translation as drug carriers (Bul et al., 2016). The phospholipid composition of liposomes is similar to pulmonary surfactants, exhibits high loading capacity for various compounds, and can improve their stability (Kawakami et al., 2000). Liposomal encapsulation of drugs has many advantages, the most important of which is to increase drug uptake and decrease drug toxicity (Courrier et al., 2002). The first liposomal product on the market for the treatment of ARDS was Alveofact^®^ (Mansour and Wu. 2009).

Small interfering RNA (siRNA) is an approach to treating respiratory diseases, including ALI/ARDS, based on nucleic acid levels (Thanki et al., 2018; Dua et al., 2019), which is primarily exerted by targeting inflammatory pathways relevant to the pathogenesis of ALI/ARDS (Qiu J et al., 2016). Liposomal nanoparticles as carriers of siRNA are more studied for the treatment of ALI/ARDS, mainly including solid lipid nanoparticles, nanostructured lipid carriers, cationic liposomes, and exosomes.

###### Solid lipid nanoparticles

The introduction of solid lipid nanoparticles (SLN) in 1991 has been known for more than 30 years (Müller et al., 2000). SLN is mainly formed by lecithin, triglycerides, and other solid-state nanocarriers (Schwarz et al., 1994) and is a promising DDS in which a biocompatible lipid matrix can encapsulate the active drug ingredient to improve the stability and solubility and can prolong the drug release time (Müller et al., 2000; Hanafy and El-Kemary, 2021). SLN contain a neutral hydrophobic core, which makes them well suited for siRNA delivery into the lung (Yonezawa et al., 2020).

The application of SLN to deliver siRNA to the lungs circumvents the stability problems associated with liposomes (Zoulikha et al., 2021). Wang et al. successfully prepared siRNA for respirable TNF-α using a thin-film freeze-drying method and encapsulated it in SLN for delivery to the lungs to silence TNF-α gene expression in cells (Wang et al., 2020). Interestingly, researchers have used aerosolized inhalation of dry powder formulations for drug administration in a manner that not only guarantees the stability of the original drug but also saves costs, reduces irritation, and is easy to administer (Hickey, 2020). This is a clear advance over previous reports by Mukherjee (Mukherjee et al., 2009). We think it is reasonable to do more trials and attempts with this approach.

###### Nanostructured lipid carriers

Nanostructured lipid carriers (NLCs) are formed by mixing liquid lipids added to the solid-state and are arguably an upgraded version of SLN (R. et al., 2003). NLCs are significantly better than SLN in drug encapsulation efficiency and long-term storage stability (Mehta, Dhanjal et al., 2020). Notably, NLCs are the least toxic nanoparticle carriers when administered *in vivo* (Muller and Keck, 2004).

It is well known that inhaled glucocorticoids are widely used in the treatment of ALI/ARDS. However, in ARDS, the aerosol is mainly deposited in the airways and can hardly reach the alveoli to exert therapeutic effects, thus not being effective for distal lung parenchymal and interstitial lung disease (Li C et al., 2017). Therefore, previous use of glucocorticoids was preferred through intravenous administration. NLCs are gradually overcoming these difficulties. NLCs encapsulated by intratracheal administration of DEX have been shown to significantly improve lung function in ALI mice and can inhibit the activation of inflammatory pathways (like NF-κB and MAPK) (Wijagkanalan et al., 2011) while also attenuating neutrophil infiltration and inflammatory cytokine expression (Wijagkanalan et al., 2008). Similar results were obtained in another study applying NLC encapsulated methylprednisolone to treat ALI rats, and no obvious liver and kidney function damage was observed with continuous administration for 2 weeks (Li N et al., 2017). Overproduction of neutrophil extracellular traps (NETs) promotes inflammation in ALI/ARDS, and NE plays an important role in forming NETs (Kaplan and Radic, 2012). As previously stated, sivelestat is an inhibitor of NE and can be used clinically in ALI/ARDS patients who present with a more severe inflammatory response. A recent study showed that inter-lamellar cross-linked multilamellar liposomal vesicles (ICMV) loaded with sivelestat (ICMV-sivelestat) were more readily taken up by neutrophils than with sivelestat alone, effectively inhibited the formation of nets, reduced the production of proinflammatory cytokines, and obviously alleviated lung injury (Okeke et al., 2020). Furthermore, NLC-loaded drugs such as omeprazole (Sun et al., 2022), EUK-134 (a superoxide dismutase/catalase mimetic) (Howard et al., 2014), and MJ-33 (a nitroxide inhibitor) (Xu et al., 2012) have all shown promising anti-inflammatory, antioxidant, and lung-protective effects in a murine model of LPS-induced ALI.

In conclusion, we have reason to believe that liposomal nanocarriers are worth recommending in improving drug loading and encapsulation efficiency, and how to find ways to improve the stability of the formulation will be a future challenge.

###### Exosomes

Exosomes (EXOs) are nano-sized vesicles secreted by various types of cells and have a lipid bilayer membrane structure with a diameter of 30–150 nm (Patil et al., 2020). They play an important role in intercellular communication, which is regarded as the natural nano-delivery system (Zhao et al., 2021). Meanwhile, the use of endogenous EXOs for ALI/ARDS treatment does not induce additional inflammatory responses.

MiRNA-26 was found to play an important role in the repair of endothelial dysfunction by human endothelial progenitor cells (EPCs), and EXOs secreted by EPCs were able to deliver miR-126 into endothelial cells leading to reduced expression of genes associated with the development of ALI/ARDS, which significantly ameliorated lung injury (Zhou et al., 2018). In another study, Zhou et al. administered EXOs (30–120 nm) of EPCs *via* the trachea to mice with LPS-induced ALI, showing beneficial effects. EXOs enriched with miR-126-3p and miR-126-5P significantly increased the expression of tight junction proteins in alveolar epithelial cells, such as zonula occludin-1 (ZO-1), occludin, and claudin (Zhou et al., 2019). EXOs containing miRNA126-5p suppressed vascular endothelial growth effector- (VEGF-) α gene expression and further improved vascular permeability. Furthermore, EXOs secreted by EPCs significantly suppressed neutrophil transmigration by improving pulmonary vascular permeability and reducing inflammatory cytokine release (Zhou et al.,^2019^).

Recently, Xu et al. isolated EXOs with a size of 50–200 nm from the lungs of phosgene-induced ALI rats, and ALI EXOs promoted MSC proliferation and increased IL-10 secretion. Furthermore, the authors identified the effective miRNA for ALI EXOs as MiR-28-5p by miRNA profiling, which activates MSCs through the PI3K/Akt signaling pathway (Xu et al., 2020).

Overall, the natural biocompatibility of EXOs compared with other exogenous carriers and the great potential to carry genetic material make them an attractive vehicle for delivery (Jafari et al., 2020).

##### Polymeric nanoparticles

Polymeric nanoparticles are generally synthesized from natural and synthetic biocompatible and biodegradable polymers with hydrophilic and hydrophobic properties, such as natural hydrophilic polymers, including proteins (e.g., albumin and gelatin) and polysaccharides (e.g., chitosan and alginate) (Chakravarty and Vora, 2020), and hydrophobic polymers, including polymethacrylates, poly(lactic-co-glycolic acid) (PLGA), polystyrene, poly(N-isopropyl acrylamide), poly(lactic acid) (PLA), poly(isobutyl cyanoacrylate) (PICA), and poly(hexyl cyanoacrylate) (Smola et al., 2008; Kaur et al., 2011; Casalini et al., 2019). These enable polymeric nanoparticles to deliver various drugs with high encapsulation efficiency, long shelf life, and protection against drug degradation (Pablo et al., 2017), making them ideal drug carriers (Mitchell et al., 2020).

The most common forms of polymeric nanoparticles mainly include two main categories: nanocapsules and nanospheres. Within these two broad categories, polymeric nanoparticles can be divided into micelles, polymersomes, and dendrimers.

###### Nanocapsule

Nanocapsules refer to solid-state colloidal particles with a size in the range of 10–1000 nm, into which the active ingredient can be encapsulated, named for their construction of a shell-like cavity in which the backbone of the particle is wrapped by a polymer membrane or polymer outer shell. High drug loading capacity and long release time are important characteristics of nanocapsules (Haggag et al., 2020).

Polymersomes are a class of synthetic vesicles that have been extensively studied over the past few decades from the synthesis of amphiphilic block copolymers (Discher et al., 2000), including polyethylene glycol (PEG) and polydimethylsiloxane (PDMS). Polymersomes can encapsulate hydrophilic molecules inside their inner cavities, whereas hydrophobic molecules can be loaded onto their surfaces (Discher and Eisenberg, 2002; Gumz et al., 2019). These polymersomes offer several advantages over conventional liposomes in their stability against dilution and prolonged drug release (Che and van Hest, 2019).

Although activated neutrophils in ALI/ARDS defend against the invasion of pathogenic microorganisms through recruitment to sites of inflammation and phagocytosis, excessive and sustained neutrophil activation exacerbates the inflammatory response (Jin et al., 2020). Delayed apoptosis and reduced clearance of neutrophils lead to severe consequences (Grudzinska et al., 2020). B-cell lymphoma- (BCL-) 2 is an anti-apoptotic protein, and venetoclax is an inhibitor of BCL-2. Free venetoclax is very difficult to be delivered to the lungs because of its extremely low water solubility, which leads to limited bioavailability and therapeutic effects. Su et al. applied synthetic amphiphilic PEG-modified poly(α-lipoic acid) nanoparticles loaded with venetoclax, and polymersomes venetoclax (P-venetoclax) increased the apoptosis of neutrophils in mice compared with free venetoclax, with better efficacy against LPS-induced ALI (Su et al., 2021).

Dendrimers are technologically advanced, synthetic, highly dendritic polymers with precisely branched three-dimensional structures (Rosen et al., 2009). The active functional groups outside of dendrimers facilitate the coupling of biomolecules to their surface, whereas small molecules and drugs can be loaded into their interior. Dendrimers can deliver multiple types of cargo but are most commonly used to deliver nucleic acids and small molecules (Palmerston Mendes et al., 2017).

In LPS-induced lung injury, mannose-modified generation 3 (G3) phosphodendrimer polymers can bind to activated dendritic cells (DCs) and ameliorate lung inflammation by inhibiting the proinflammatory cytokine TNF-α in DCs (Blattes et al., 2013). Recently, Li et al. developed G3 phosphodendrimers with a hydrophobic alkyl chain (C11H23) as the core and surface modification with bisphosphate groups followed by hydrolysis to yield tyramine containing sodium dimethyl phosphonate (TBP) terminal anionic amphiphilic phosphodendrimers (C11G3-TBP). Encapsulation of Cur by C11G3-TBP resulted in a C11G3-TBP/Cur mixture, which could effectively scavenge ROS, inhibit proinflammatory cytokines (TNF-α, IL-1β, and IL-6) release, block the NF-κB signaling pathway in M1 type alveolar macrophages, and enable efficient repolarization of M1 type alveolar macrophages to the anti-inflammatory M2 type to exert anti-inflammatory and antioxidant effects (Li et al., 2022).

###### Nanosphere

Nanospheres are solid-state polymeric spheres with backbone particles as entities. Drugs can be encapsulated in the middle or on the surface of the sphere, which is the most significant difference from nanocapsules. Micelles are nanospheres with a lipophilic inner core and a hydrophilic outer shell, which facilitates the entrapment of poorly soluble drugs and improves drug release time. Micelles can be loaded with various cargoes and have been used in clinical trials to deliver therapeutic drugs.

Micelles refer to the self-assembly of molecules in an aqueous solution to form stable, glue-like aggregates with diameters in the range of 10–100 nm in an ordered arrangement (Wang J et al., 2015). Currently, micelles are mostly constructed of amphiphilic block copolymers, named polymeric micelles (PMs). Polymeric micelles consist of a lipophilic core and a hydrophilic outer shell, which are used to encapsulate hydrophobic drugs, whereas a hydrophilic outer shell prevents aggregation and precipitation of the micelles. This structure allows the micelles to maintain good stability (Wang Z et al., 2015). In preclinical and clinical studies, the use of polymeric micellar formulations can greatly alleviate side effects and greatly improve the quality of life of patients (Danson et al., 2004).

Polyethylene glycol (PEG) has become the most commonly used hydrophilic block material of PMS due to its high safety in humans (Hwang et al., 2020). Cur is a very hydrophobic drug, and its exertion is highly dependent on the local microenvironment (Lübtow et al., 2019). PEG-modified PLGA was used to prepare Cur-loaded nanospheres with good biocompatibility and biodegradability, which improved the bioavailability of Cur upon ALI (Jin et al., 2019). Heme oxygenase- (HO-) 1 has well-established anti-inflammatory and antioxidant properties and is a therapeutic target for various inflammatory diseases, including ALI/ARDS (Oh et al., 2019). Based on this, Kim et al. loaded Cur into cholesterol conjugated Polyacrylamide (PamChol) micelles, obtained a Cur-loaded mixture (PamChol-Cur), and then fused it with HO-1 expression plasmid DNA (p-DNA) *via* charge interaction to form the pDNA/PamChol-Cur complex. Luciferase assay showed that the *in vitro* plasmid DNA delivery efficiency of pDNA/PamChol Cur delivered to lung epithelial cells was significantly higher than that of the pDNA group and exerted anti-inflammatory effects by inhibiting the nuclear translocation of NF-κB (Kim et al., 2019).

Choi et al. (2021) used cholesterol coupled to histidine and arginine grafted polyamidoamine (PamHR) to form micelles (PamHRchol), followed by a reaction with glycyrrhizic acid (GA) to produce mixed dendritic micelles (PamHRchol/GA), and the HO-1 gene was introduced into the lungs of ALI mice as a therapeutic gene. Dendrimeric micelles have higher gene delivery efficiency compared to free GA. Both *in vitro* and *in vivo* results demonstrated that PamHRchol/GA micelles decreased the inflammatory factor TNF-α expression, with obvious anti-inflammatory effects. Other block materials, such as polyethyleneimine (PEI) (Su et al., 2022), have also been reported to modify PMS with significantly improved lung targeting ability.

It is well known that neutrophil overactivation during ALI/ARDS releases cytotoxic substances, such as ROS and NE, which can aggravate the severity of ALI/ARDS. Brannon et al. developed a ∼ 1 μM of salicylic acid-based poly(anhydride ester) nanosphere, called poly(salicylic acid) (poly-A). Poly-A nanospheres were injected into mice *via* tail vein injection 18 h after a lung infection. The results showed that untreated ALI/ARDS mice died within 48 h, whereas 80% of the mice receiving poly-A injection could survive to a week. In addition, poly-A particles enter the neutrophils and are rapidly degraded, and the released salicylic acid acts directly on the neutrophils and significantly alleviates the inflammatory response (Brannon et al., 2022). In recent years, inhaled nanospheres developed to prolong drug action time have gained increasing attention in treating ALI/ARDS. In paraquat-induced ALI, the administration of porous selenium (Se) @ SiO_2_ nanospheres *via* inhalation significantly decreased the levels of ROS, malondialdehyde, NF-κB, p-NF-κB, TNF-α, and IL-1β in lung tissue. It significantly increased the levels of glutathione and superoxide dismutase (Yong et al., 2017). This may be supported by the porous Se@SiO_2_. The protective effect of nanospheres on Se was only released after it reached the lung safely to avoid being degraded prematurely (Liu et al., 2016). Moreover, the nanospheres can enter alveolar macrophages without activating them, thus prolonging the time for drugs to be cleared (Courrier et al., 2002).

##### Inorganic nanoparticles

Inorganic materials such as gold, silver, calcium, iron, and silica have been used to synthesize nanostructures involved in the delivery of various drugs. Inorganic nanoparticles mostly have good biocompatibility and stability, which can meet some special applications that cannot be reached by organic materials (Mitchell et al., 2020). These advantages make it widely used in the treatment of ALI/ARDS.

Among inorganic nanoparticles, mesoporous silica nanoparticles are considered the best carriers for drug delivery (Zeng et al., 2017; García-Fernández et al., 2021). Because its outer surface can be decorated with a biomolecular gating system (also referred to as a “nano-valve”), the release of drugs can be controlled upon encountering stimuli, such as inflammation, a characteristic that is significantly superior to nanoparticles constructed with liposomal and polymeric carriers, as the latter typically release encapsulated drugs out through carrier degradation (Kamaly et al., 2016; Shi et al., 2020). Gated mesoporous silica nanoparticles were surface-coated with a peptide that could target TNFRⅠ expressed in macrophages after encapsulating DEX inside the silica nanoparticles and injecting it into LPS-induced ALI mice *via* the tail vein, compared with the group treated with DEX injection alone, its controlled release of DEX, and significant reduction levels of inflammatory factors (TNF- α, IL-6, and IL-1β) (García-Fernández et al., 2021). Yuan et al. (2021) administered iron curcumin nanoparticles (Fe-Cur) NPs to ALI mice by tracheal inhalation and intravenous injection, respectively, and both modes of administration could downregulate several important inflammatory cytokines (TNF-α, IL-1β, and IL-6) levels, suppressing the expression of NLRP3 inflammasome and suppressing NF-κB signaling pathway activation, but effectively achieving ROS scavenging in lung tissue. Inorganic NPs also have the potential to participate in regulating macrophage polarization. Studies have found that CeO_2_ has great antioxidant and anti-inflammatory capacities. However, it has limited its use due to its slow clearance in the body and the cumulative generated toxic reactions (Yokel et al., 2012). Serebrovska et al. (2017) loaded CeO_2_ on the surface of SiO_2_ nanoparticles, forming a mixture of (CeO_2_@SiO_2_) and reduced the generation of ROS and the expression of pro-inflammatory cytokines (TNF-α, IL-6) in lung tissues, which was easier to be cleared from the body. Mitochondria are involved in various physiological processes, including redox reactions and programmed cell death. The Se@SiO_2_ nanoparticles developed by Wang et al. significantly increased the resistance of airway epithelial cells to LPS-induced oxidative damage. Moreover, they can achieve the purpose of protecting cells by maintaining mitochondrial function and dynamics [mitochondrial dysfunction can contribute to the development of inflammatory lung diseases (Kellner et al., 2017)]. In the animal model of LPS-induced ALI, Se@SiO_2_ nanoparticle pretreatment attenuates lung inflammatory responses and diffuses damage to lung tissue (Wang et al., 2020).

A nanoparticle (P12) with anti-inflammatory properties made of hexapeptide-coated gold nanoparticles (GNPs) is administered *via* nasal inhalation. Interestingly, in an ALI mouse model, P12 could increase alveolar M2 macrophages (elevated IL-10 expression) and decrease M1 macrophages in BALF and lung tissue, thus effectively protecting the lung from injury and promoting inflammation resolution (Wang L et al., 2020). Interestingly, p12 could be modified by cigarette smoke extract (CSE), which significantly enhanced the inhibitory activity of p12 on TLR4 mediated inflammatory responses both *in vitro* and *in vivo*, and soluble components in CSE may play a major role in modifying p12 with stronger anti-inflammatory activity (Gao et al., 2019a). Furthermore, p12 was observed to have GNP nuclear size-dependent anti-inflammatory effects in the LPS-induced ALI mice model. Specifically, p12 (G20) with a GNP core of 20 nm is superior in prolonging the survival of mice, reducing lung inflammation, and diffusing alveolar damage compared to p12 (G13) with a GNP core of 13 nm (Gao et al., 2019b).

Generally, inorganic nanoparticle carriers have many advantages, such as uniform pore size, long retention time, and good biocompatibility and stability (Yang et al., 2012; Pascual et al., 2017). Nevertheless, some deficiencies should also be focused on: a large number of studies have shown that inorganic nanoparticles (titanium dioxide (Sun et al., 2012), gold, and silver (Bachand et al., 2012; Kheiralla et al., 2014) can produce cytotoxic effects, promote the accumulation of ROS, and lead to lung inflammation. In addition, solubility also limits their widespread use (Arias et al., 2018). Therefore, inorganic nanoparticles may not be recommended for routine use as vehicles for pulmonary drugs, and future research directions should focus on reducing toxicity and improving solubility.

##### Natural carrier nanoparticles

Natural carrier nanoparticles are mainly used for biological therapy of ALI/ARDS, including antibodies, peptide or protein vaccines, gene vaccines, and *in vivo* gene therapy. Herein, we mainly introduce nanobodies, cell membrane nanobodies, and DNA nanoparticles when used as drug carriers to treat ALI/ARDS.

###### Nanobodies

Nanobodies (NBs) are a class of natural antibodies reported almost 30 years ago (Hamers-Casterman et al., 1993). NBs are a subset of light chain missing heavy-chain antibodies found in the blood of camelid (camel, alpaca, *etc.*) animals that contain only one heavy chain variable region (VHH). Interestingly, VHHs, the key crystals in which NBs function, are approximately 2.5 nm in size and retain the ability to bind antigens, which are the smallest known antigen-binding crystals (Muyldermans, 2013).

The application of NBs is mainly focused on the study of lung injury caused by viral respiratory diseases, such as the anti-respiratory virus nanobody ALX-0171 (manufactured by ABLYNX company) formulated in an inhaled dosage form with properties against the respiratory syncytial virus (RSV) (Detalle et al., 2016). Compared with conventional antibodies, NBs have the advantages of being easily permeable to the blood–brain barrier, low manufacturing cost, and high affinity (Kolkman and Law, 2010). However, their clearance through the kidney is too fast to result in low drug concentrations at the lesion (Jovčevska and Muyldermans, 2020). Overall, NBs overcome the disadvantages of low stability and uneasy preservation of traditional antibodies. They also have the advantage of small molecule drugs, making them an emerging force in a new generation of genetically engineered medicine.

###### Cell membrane biomimetic nanomedicine

Cell membrane nanotechnology, also known as cell membrane biomimetic nanotechnology, has partly revolutionized drug delivery strategies in terms of stability, biodistribution, safety, and toxicity of biomimetic carriers (Yoo et al., 2011). Compared with traditional methods, biomimetic carriers have the function of natural cell adhesion molecules, which makes them a novel type of DDS.

Red blood cells (RBCs), the most abundant circulating cells in the blood, have been widely applied in the delivery of drugs because of their biocompatibility and long circulating half-life. Researchers have used positively charged chitosan prepared nanoparticles (CSNPs) to combine with negatively charged erythrocyte membrane to form erythrocyte membrane nanocarriers, and methylprednisolone sodium succinate was loaded on the erythrocyte membrane nanocarrier. The RBC membrane nanocarrier group significantly inhibited the production of inflammatory cytokines such as TNF-α and IL-6 and the expression of IL-1β and caspase-1 in the lung tissue of ALI/ARDS and prolonged the drug release time (Ding et al., 2022).

During the inflammatory process in ALI/ARDS, the endothelial system passing the NF-κB signaling pathway promotes intercellular adhesion molecule 1 (ICAM-1) expression, binding to integrins on the neutrophil membrane β2 interaction involved in the inflammatory response (Schymeinsky et al., 2009). Gao et al. created a nanovesicle-based DDS using nitrogen cavitation technology. This system destroys activated neutrophils, thus obtaining cell membrane nanovesicles. Intravital microscopy studies in living mice have shown that the obtained cell membrane nanovesicles have a full complement of integrins because the β2 targeting molecules can selectively bind to sites of inflammation in the vasculature. The application of cell membrane nanovesicles loaded with TPCA-1, an NF-κB inhibitor, significantly attenuated acute lung inflammation in mice (Gao et al., 2016).

Extracellular vesicles (EVs) are nanoscale vesicles secreted by cells. Platelets are associated with pulmonary neutrophil accumulation, leading to altered alveolar-capillary permeability and pulmonary edema formation (Rossaint et al., 2014). Extracellular vesicles (PEVs) derived from platelets are a type of cell-based drug delivery vehicle. In the mice model of ALI, when loaded with TPCA1, PEVs significantly inhibited inflammatory cell infiltration in the lungs and significantly alleviated the local cytokine storm (Ma et al., 2020).

###### DNA nanodevice

DNA is a natural biomaterial that has become an ideal drug carrier for biomedicine due to its biocompatibility, biodegradability, and programmability. Due to their programmability and strictly following the principle of complementary base pairing, DNA nanodevices can be assembled into different geometries to cross the cell membrane into cells to exert their functions (Guan et al., 2021). DNA nanodevices with artificial intelligence (AI) have attracted great interest, which may open a new chapter in precision medicine for ALI/ARDS.

In general, we need to reprogram DNA complementary sequences by AI so that they can be assembled *de novo* from covalently bonded carbon chains (Nummelin et al., 2018), further forming more nanoscale devices that are then integrated into living cells for study using synthetic biology approaches (Douglas et al., 2012). In addition to being used as a small molecule or drug carrier alone, DNA nanodevices can be combined with other nanomaterials, such as metallic nanoparticles, for better therapeutic outcomes. DNA-based nanodevices have been used for cancer immunotherapy (Cui et al., 2021; Li and Fan, 2021; Liu Y et al., 2021). Cytosine phosphate guanine (CPG) methylation is considered a molecular level biomarker for lung cancer. Recently, researchers used the cooperative *in situ* assembly of G-quadruplex DNAzyme nanowires to quantify different CpG methylation targets of different human cancer cells and found that this DNA nanodevice could discriminate the methylated expression of CPGs in lung cancer and precancerous tissues (Wang et al., 2022). Moreover, DNA nanodevices can be rapidly degraded and eliminated *in vivo*, reducing the risk of potential toxicity (Jiang et al., 2016).

The newly developed DNA nanorobot achieved dynamic mechanical properties based on DNA origami technology, which can carry drugs to reach the lesion site following the designed path (Zhang et al., 2019), providing potential application prospects for better-regulated drug release.

## Discussion and conclusions

ALI/ARDS is a common critical illness in respiratory care units with a huge public health burden. With the use of noninvasive positive-pressure ventilators and medications, the mortality of ARDS has declined from 80% in the 1960s to around 30% at present (Bellani et al., 2016). Despite tremendous advances in the prevention and treatment of ARDS, ∼3 million patients with ARDS per year remain the number one cause of ICU management and MV (Bellani et al., 2016), and the mortality rate of ARDS remains unacceptably high (Chen et al., 2019).

The poor performance of the ARDS management can be attributed to the following reasons (Brenner, 2017; Brenner et al., 2018): first, ARDS presents complex etiology and heterogeneity, which makes it unlikely to show curative effect depending on one pathway alone; second, patients with ARDS are critically ill, most merging with multiorgan failure, thus being unable to tolerate side effects brought by drug off-target; and third, as the alveoli are covered by a large volume of highly proteinaceous fluid in ARDS, drugs are hardly effectively absorbed and utilized.

As discussed separately, the solution to the first limitation is to stratify the etiology of patients and clarify the pathophysiological mechanism and inflammatory response pathway of ARDS. Accordingly, individualized drug treatment is implemented. Moreover, we have made some advances in some pre-clinical or clinical status, such as KGF in mitigating lung endothelial and epithelial injury, anti-platelet or anti-cytokine in suppressing inflammation, and target RAAS in clearing edema fluid (Table 1).

Unfortunately, due to the inability caused by previously mentioned second and third limitations, dozens of them mostly fail in ARDS clinical trials (S et al., 2015). These reduced expectations reflect the necessity of the way of effective drug delivery. The current main challenges facing successful drug delivery in ARDS are the pulmonary clearance mechanisms, the metabolic degradation of drugs, and the control of drug deposition sites and rates (Cepkova and Matthay, 2006; El-Sherbiny et al., 2015; García-Fernández et al., 2021). To solve it, directly targeted delivery of drugs to the lungs is now an attractive prospect as the large surface area and abundant blood vessels in the alveolar region and avoidance of first-pass metabolic clearance (Courrier et al., 2002; Pilcer and Amighi, 2010; Onoue et al., 2015). Therefore, in order to achieve this prospect, the researcher’s concern has turned to the following points: how to ensure the precise deposition of drugs and exert the therapeutic effect? By what route?

Accordingly, targeted pulmonary nano-DDS may be a useful approach in ARDS pharmacologic therapy and gaining notice in recent years. It has unique physicochemical characteristics, including prolonged drug release time, improved drug solubility, limited systemic exposure, reduced toxic side effects of drugs, increased bioavailability, and safety (Koo et al., 2005; Patton and Byron, 2007; Longfa et al., 2018; Yoshida et al., 2020). For example, NO, with a half-life of only a few seconds, can be easily diluted and metabolized *in vivo*. However, it can be sustained-release nanoparticles *via* nanotechnology, resisting unnecessary degradation of drug molecules, greatly extending its action time, and contributing to its use in the treatment of ARDS (Akmal and Hasan, 2008; Rana, 2021; Shurbaji et al., 2021). Meanwhile, nanoparticles, especially nanospheres, can enter macrophages without activating macrophages, effectively overcoming the clearance caused by alveolar macrophage uptake in the alveolar region (Courrier et al., 2002; Patton and Byron, 2007; Mansour et al., 2009).

After processing different drugs through nanotechnology to ensure their precise effects, the route to deliver them to the lungs become clear: inhalation. Compared with the other administration route, this non-injectable route has many advantages. On the one hand, drugs can be rapidly absorbed and avoid the first-pass metabolic clearance through the alveolar area and blood vessels (Courrier et al., 2002; Pilcer and Amighi, 2010; Onoue et al., 2015). On the other hand, direct delivery of low concentrations of drugs to the target site produces the greatest accumulation of therapeutic effects through minimum doses and reduces the risk of toxicity and systemic exposure (Cipolla et al., 2013). Drug delivery to the lungs *via* inhalation has emerged as a useful delivery route for the treatment of ARDS since 2017 (Maselli et al., 2017), including inhalable nanocrystalline aerosols (INA) (Chiang et al., 2009), inhalable nanocrystal-based composite microparticles (INCM) (Luo et al., 2020), inhalable nanocrystal based adhesive microparticles (Liu et al., 2018), and inhalable mucus penetrating nanocrystals (IMN) (Costabile et al., 2020), all of them exhibiting favorable nebulization properties.

Although aerosolized inhaled nanomedicine shows promising prospects for its better future clinical application, the objective evaluation of the potential pitfalls is warranted. First, although the toxic effects produced by nanomaterials on cells are poorly reported, some drugs applied *in vitro* may not be suitable *in vivo*. For example, polycyanoacrylate nanoparticles are highly toxic to airway epithelial cells, rendering them unavailable as vehicles for the treatment of ARDS (Lherm et al., 1992; Wong et al., 2020). Second, the disrupted mucociliary system in ARDS results in the inability of clearance, causing nanoparticles to stock in the respiratory tract and lung. This excessive dose accumulation of nanoparticles ultimately results in nanoparticle overload and inflammatory responses (Oberdorster, 1995; Ryan et al., 2013; Haque et al., 2018). In addition, aerosolized inhaled formulations have drawbacks, such as the short duration of clinical effects and the requirement for frequent drug administration (Yoshida et al., 2020; Shurbaji et al., 2021). Meanwhile, nanoparticles are too small and may easily be exhaled after inhalation, thereby greatly reducing their deposition in the airways (Patton and Byron, 2007). These two reasons lead to limited lung absorption of some biodegradable nanoparticles (Patton, 1996; Miller et al., 2017). Although aerosolization into the lungs in the form of nanosuspensions more or less modifies the issue of being exhaled, it can easily cause the nanosuspensions to agglomerate or be lost during aerosolization (Paul et al., 1998). Therefore, it is important to further elucidate the long-term drug safety of nanoparticulate drugs and the kinetics of drug carriers (Forbes et al., 2014).

Besides, natural herbs and their extracts have unique advantages in treating ARDS, such as lower toxicity and side effects, low price, and promising therapeutic potential. If researchers conduct clinical research on natural medicine therapy according to the evidence-based standards of modern randomized controlled trials, we may discover more valuable treatment pharmacology.

ARDS is a syndrome characterized by heterogeneity. With a better understanding of the pathophysiology, more therapy drugs targeting different phases are needed. Among them, nanomedicine offers new opportunities and options for treating ARDS and is a promising therapeutic tool. Today, where COVID-19 is still ravaging, pharmacologic therapies, especially the potential of nanomedicine as a powerful delivery system for the treatment of ARDS, deserve further research.
